# Integrated analyses reveal the response of peanut to phosphorus deficiency on phenotype, transcriptome and metabolome

**DOI:** 10.1186/s12870-022-03867-4

**Published:** 2022-11-14

**Authors:** Qi Wu, Liyu Yang, Haiyan Liang, Liang Yin, Dianxu Chen, Pu Shen

**Affiliations:** Shandong Peanut Research Institute/Key Laboratory of Peanut Biology, Genetics & Breeding, Ministry of Agriculture and Rural Affairs, Shandong Academy of Agricultural Sciences, 126 Wannianquan Road, Qingdao, 266100 China

**Keywords:** *Arachis hypogaea*, Phosphorus deficiency, Transcriptome, Metabolome, miRNAs, Lignin

## Abstract

**Background:**

Phosphorus (P) is one of the most essential macronutrients for crops. The growth and yield of peanut (*Arachis hypogaea* L.) are always limited by P deficiency. However, the transcriptional and metabolic regulatory mechanisms were less studied. In this study, valuable phenotype, transcriptome and metabolome data were analyzed to illustrate the regulatory mechanisms of peanut under P deficiency stress.

**Result:**

In present study, two treatments of P level in deficiency with no P application (–P) and in sufficiency with 0.6 mM P application (+ P) were used to investigate the response of peanut on morphology, physiology, transcriptome, microRNAs (miRNAs), and metabolome characterizations. The growth and development of plants were significantly inhibited under –P treatment. A total of 6088 differentially expressed genes (DEGs) were identified including several transcription factor family genes, phosphate transporter genes, hormone metabolism related genes and antioxidant enzyme related genes that highly related to P deficiency stress. The Gene Ontology (GO) and Kyoto Encyclopedia of Genes and Genomes (KEGG) enrichment analyses indicated that 117 genes were annotated in the phenylpropanoid biosynthesis pathway under P deficiency stress. A total of 6 miRNAs have been identified significantly differential expression between + P and –P group by high-throughput sequencing of miRNAs, including two up-regulated miRNAs (ahy-miR160-5p and ahy-miR3518) and four down-regulated miRNAs (ahy-miR408-5p, ahy-miR408-3p, ahy-miR398, and ahy-miR3515). Further, the predicted 22 target genes for 6 miRNAs and *cis*-elements in 2000 bp promoter region of miRNA genes were analyzed. A total of 439 differentially accumulated metabolites (DAMs) showed obviously differences in two experimental conditions.

**Conclusions:**

According to the result of transcripome and metabolome analyses, we can draw a conclusion that by increasing the content of lignin, amino acids, and levan combining with decreasing the content of LPC, cell reduced permeability, maintained stability, raised the antioxidant capacity, and increased the P uptake in struggling for survival under P deficiency stress.

**Supplementary Information:**

The online version contains supplementary material available at 10.1186/s12870-022-03867-4.

## Background

Peanut (*Arachis hypogaea* L*.*) is an important oil crop and nutritious food, which is rich in high-quality plant protein, oil and lipid compounds. Peanut ranks the fourth in oil crops and third in human protein sources all over the world [1]. Peanut shows not only better yield but also higher quality when it is planted on suitable soil nutrition condition [2]. Phosphorus (P) is one of the most essential mineral nutrients for all crops due to its critical roles in plant physiological and biochemical processes [3]. Peanut always requires large amounts of P, which can participate directly in both the process of photosynthetic apparatus establishment and photosynthetic carbon assimilation. P also plays an important role in the formation of peanut yield and quality, in the transformation and accumulation of fats, proteins, phospholipids, fatty acids and nucleotides. Regularly, the content of P in most plants generally accounts for 0.1%–0.5% of dry weight (DW), while that in peanut kernel ranges from 1%–2% DW among different peanut varieties [4]. However, P deficiency is widespread in many peanut field soils with the low availability and total P content [5]. Low soil available P is one of the important factors affecting the growth of peanuts [6]. The serious lack of available P in soil is not only a limiting factor for the development of agricultural production in the world but also an important bottleneck for the sustainable development of the peanut industry.

Under P deficiency stress, crop phenotype, physiology, biochemistry and even microscopic levels have adaptive changes, which are ultimately reflected in plant growth and yield [7, 8]. The root system is the main organ for P absorption while both morphology and architecture change to adapt to the P deficiency soil environment. The number of lateral roots and the density and length of root hairs are increased to expand the contact areas between the root system and the soil, which is very important to raise extraction efficiency at low P level [9, 10]. In addition, roots can also secrete ACP to activate insoluble organic phosphorus into absorbable H_2_PO_4_^–^ or HPO_4_^2–^ in soil to achieve more phosphorus absorption for growth [11–14]. Moreover, P in deficiency inhibits the photosynthesis system and lead to excessive accumulation of ROS, which causes damage to DNA, proteins and lipids [15, 16]. Meanwhile, plants activate their own internal antioxidant system to protect against ROS damage [17], including many enzymatic components, such as SOD, POD, and CAT. P is crucial for photosynthesis because most steps on photosynthesis require P nutrition [18]. At the physiological level, P in deficiency reduces the ability to utilize sunlight, as it can cause photosystem damage and photosynthetic rate decrease [19, 20]. Consequently, the inhibition of photosynthesis leads to a reduction in biomass and limits plant growth and development [21].

However, at the molecular and metabolic level, the regulation mechanisms under P deficiency still remains incomplete. Combined omics analyses provides integrated and focused clues for intensive and comprehensive explanation of plant response mechanism. Transcriptome analysis under P deficiency stress will be an efficient way to identify candidate genes and putative metabolites synthesis involved in low P stress response. MicroRNAs (miRNAs) can regulate diverse growth and development programs under stress conditions through post-transcriptional repression by cleaving target genes mRNA in plant [22–26]. Metabolites were the production of genes expression and transcriptional regulation, which would directly affect the phenotype of stress response. Metabolism can provide both type and quantity of endogenous metabolites. When joint analysis with transcriptome results, it was more helpful to elucidate relative genes, miRNAs, and focus on metabolite synthesis and regulated pathways [27, 28].

In present study, we designed experiments in a hydroponic system to examine the effect of P deficiency on the phenotype, transcriptome and metabolome properties of peanut development. To investigate the effect of P deficiency on peanut development, two groups of plants grown on –P and + P application of Hoagland’s nutrient solution were carried out, respectively. The research tried to find the differences in plant phenotype and molecular mechanisms under low P condition. It is of great significance to explore the adaptive adjustment of P absorption and utilization under P deficiency stress in peanut.

## Results

### Growth and development inhibition of peanut under P deficiency stress

**Fig. 1:**
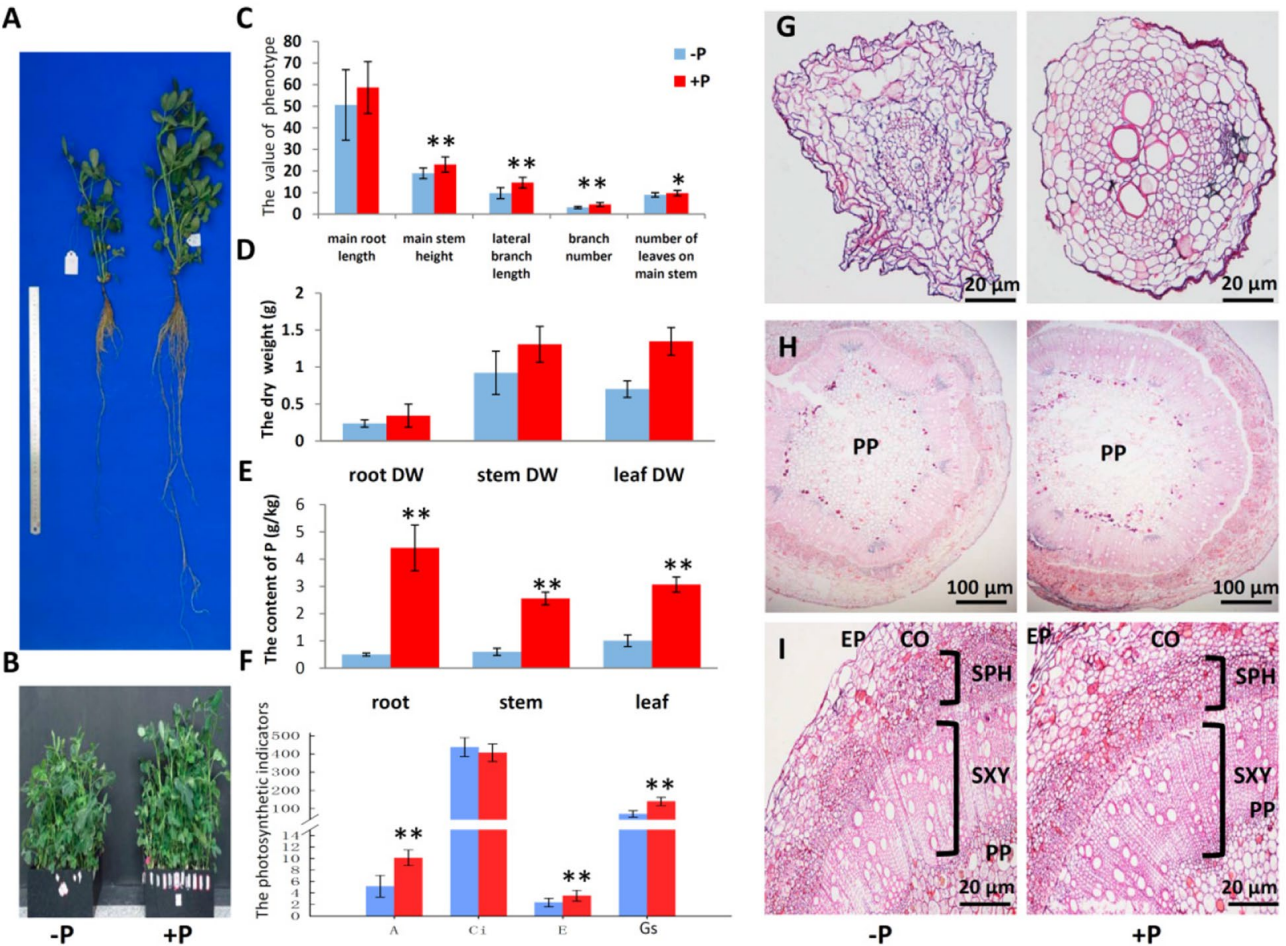
Response of peanut plant to the different phosphorus supplies (–P, 0 mM Pi and + P, 0.6 mM Pi). **A** and **B** Differences of plant in two P level treatments. **C** Differences of phenotype indicators of peanut plant. **D** Differences of root, stem, and leaf dry weight. DW, dry weight. **E** P content in different tissues between two P level groups. **F** Differences of photosynthetic indicators in two P level treatments. A, net photosynthetic value; Ci, intercellular CO_2_ concentration; E, transpiration rate; Gs, stomatal conductance. Column denoted mean value, and bars indicate standard errors. **G** Histochemical sections of primary root apex in two P levels. **H** and **I** Histochemical sections of the basal of main root in two P levels. PP, parenchyma pith; SXY, secondary xylem; SPH, secondary phloem; CO, cortex; EP, epidermis. *, *P* < 0.05. **, *P* < 0.01

The phenotypes of plant in two P level Hoagland nutrient solutions for 60 days were investigated. Obvious variation was found between two groups for all tested growth parameters. The effect of P deficiency stress was significant for the inhibition of growth and development in peanuts. The plants under –P treatment was significantly lower than that under + P treatment (Fig. 1A, B). The main root length, main stem height, lateral branch length, number of branches and number of leaves on the main stem decreased by 13.8%, 17.6%, 33.3%, 30.5% and 8.7%, respectively, when P was deficient (Fig. 1C). DW of roots, stems and leaves under + P treatment were all heavier than that under –P treatment by 44.6%, 41.9% and 92.0%, respectively (Fig. 1D). The WinRHIZO root analysis system was used to scan the root morphologies of two treatments. The data showed that P deficiency stress reduced root development compared with the P-sufficiency condition (Supplemental Table S1). Under + P treatment, mean value of total length (cm), mean value of total surfArea (cm^2^), mean value of total volume (cm^3^), and mean value of total tips were increased by 24.0%, 28.2%, 16.3%, and 25.7% compared with P deficiency stress condition.

To detect the plant P content, three samples were randomly taken from each treatment. Under –P treatment, the P content of the peanut root, stem, and leaf were 0.5, 0.6, and 1.0 g kg^−1^ DW, respectively, which were significantly lower compared with that under + P treatment (Fig. 1E). After P supply, the P content values of the three parts were significantly increased to 4.4, 2.6, and 3.1 g kg^−1^ DW, respectively. Compared with –P treatment, the total P content was increased under + P treatment by 8.8, 4.3 and 3.0 times, respectively. This result indicated that the P content had a great impact on the whole plant and specially had the greatest impact on the roots. Additionally, the accumulative P amount (P content × DW) was detected. Compared with –P treatment, the cumulative P content in root, stem and leaf under + P treatment were 12.07, 6.24 and 6.15 times, respectively.

For the effect of P on photosynthesis, 4 photosynthetic indexes were carried out on the third fully expanded upper leaves of each plant by the CIRAS-3 portable photosynthesis system at 60 days after planting. The A, E and Gs values under + P treatment were extremely significantly higher than that under –P treatment (*P*< 0.01), but there was no significance among the Ci value. Under P deficiency stress, A of peanut functional leaves was 5.2 μmol CO_2_ m^–2^ s^–1^, E was 2.4 mmol H_2_O m^–2^ s^–1^, Ci was 437.0 μmol CO_2_ mol^–1^, and Gs was 70.9 mol H_2_O m^–2^ s^–1^. P application significantly improved the net photosynthetic rate by 95.0%, the transpiration rate by 49.8%, the stomatal conductance by 96.0%, whereas decreased the intercellular carbon dioxide concentration by 7.0% (Fig. 1F).

From the cross-section of primary root apex under + P treatment, the structure was obvious with small epidermal cells, a wide cortical area and a vascular cylinder in the internal part. The red lignified cell wall under P deficiency stress was lighter in red accompanied by thinner, irregular cell shape. And many small cells were observed inside, indicating that it was still in the process of growth and development while Under + P treatment, the cell wall was darker red and the cell wall was thicker, the cell shape was regular (Fig. 1G). The root system first perceived the P deficit of soil. From the cross sections of the basal roots under two treatments, the secondary structure could be seen to be typical of leguminous coal. The PP was composed of parenchyma cells. The main function of the pith was to store essential nutrients such as water and sugar. The PP zone of the control group was nearly round, while the shape under –P treatment was irregular. With seriously P deficiency stress, the overall shape of PP cells decreased and became tighter with fewer intervals (Fig. 1H). The SXY was consisted of broad vessels. Both SXY and SPH under + P treatment were both apparently wider and more orderly than that under –P treatment. The CO cells composed of parenchyma cells were few, numerous, closely arranged and small in space under + P condition, and the structure was still regular and orderly. However, the cortical parenchyma cells under –P stress were larger, fewer in number and loosely arranged, and some were even broken, which were apparently at the same situation as the EP (Fig. 1I).

**Fig. 2:**
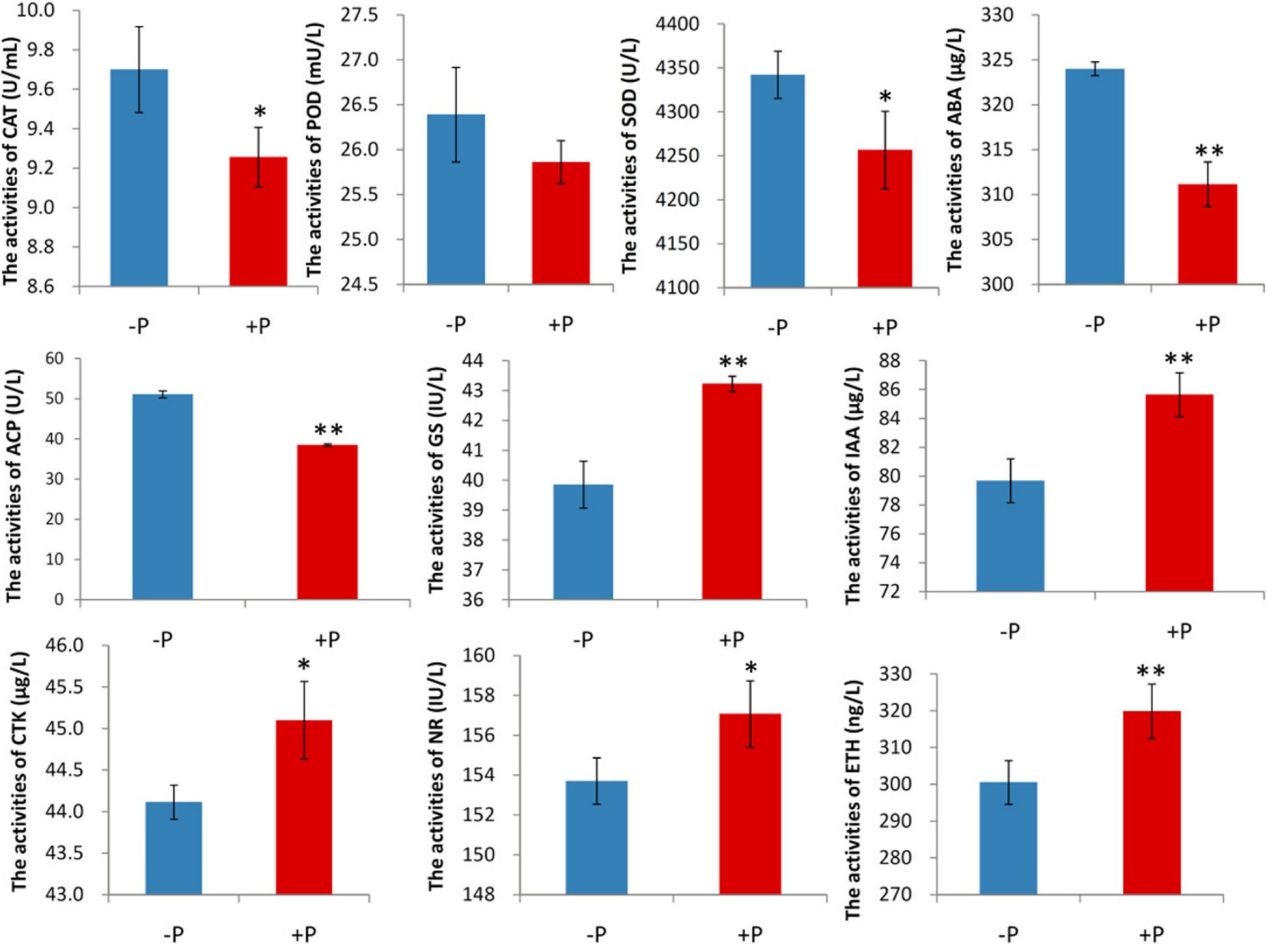
Effect of phosphorus on activities of related enzymes and hormones in peanut roots. * indicates *P* < 0.05. ** indicates *P* < 0.01

P deficiency stress changed the activities of enzymes related to antioxidants, plant growth and nutrient absorption. The activities of 10 enzymes in the peanut roots were detected by using ELISA methods (MDBio, Taipei, China). The P deficiency stress enormously increased the activities of CAT, SOD, ABA and ACP compared to that in + P condition, respectively. Simultaneously, –P stress obviously decreased the activity of GS, IAA, CTK, NR, and ETH. The activity of POD was also increased under P deficiency condition but with no significance (Fig. 2). The activities of enzymes related to antioxidants were also detected in leaves. Under –P treatment, the activities of GS and ETH were markedly increased, but the activities of ABA and NR were obviously decreased compared with that under + P treatment. Moreover, the activities of CAT, POD, SOD, and IAA under –P treatment were slightly higher than that under + P treatment with no significance in leaves (Supplemental Fig. S1).

### Transcriptome analysis of peanut roots under P deficiency stress

A total of 31.00 GB of high-quality clean data were obtained with the 5.16 GB average clean data of each sample, 94.73% at the Q30 level, average GC% at 44.96%, which suggested that the sequencing was highly accurate and reliable (Supplemental Table S2). The genome of the peanut cultivar Shitouqi (PGR: http://peanutgr.fafu.edu.cn/Download.php) was used as the *Arachis hypogaea* L. reference genome. The clean reads of each sample were mapped with the reference genome, and the mapped percentages ranged from 86.05% to 91.88%.

**Fig. 3:**
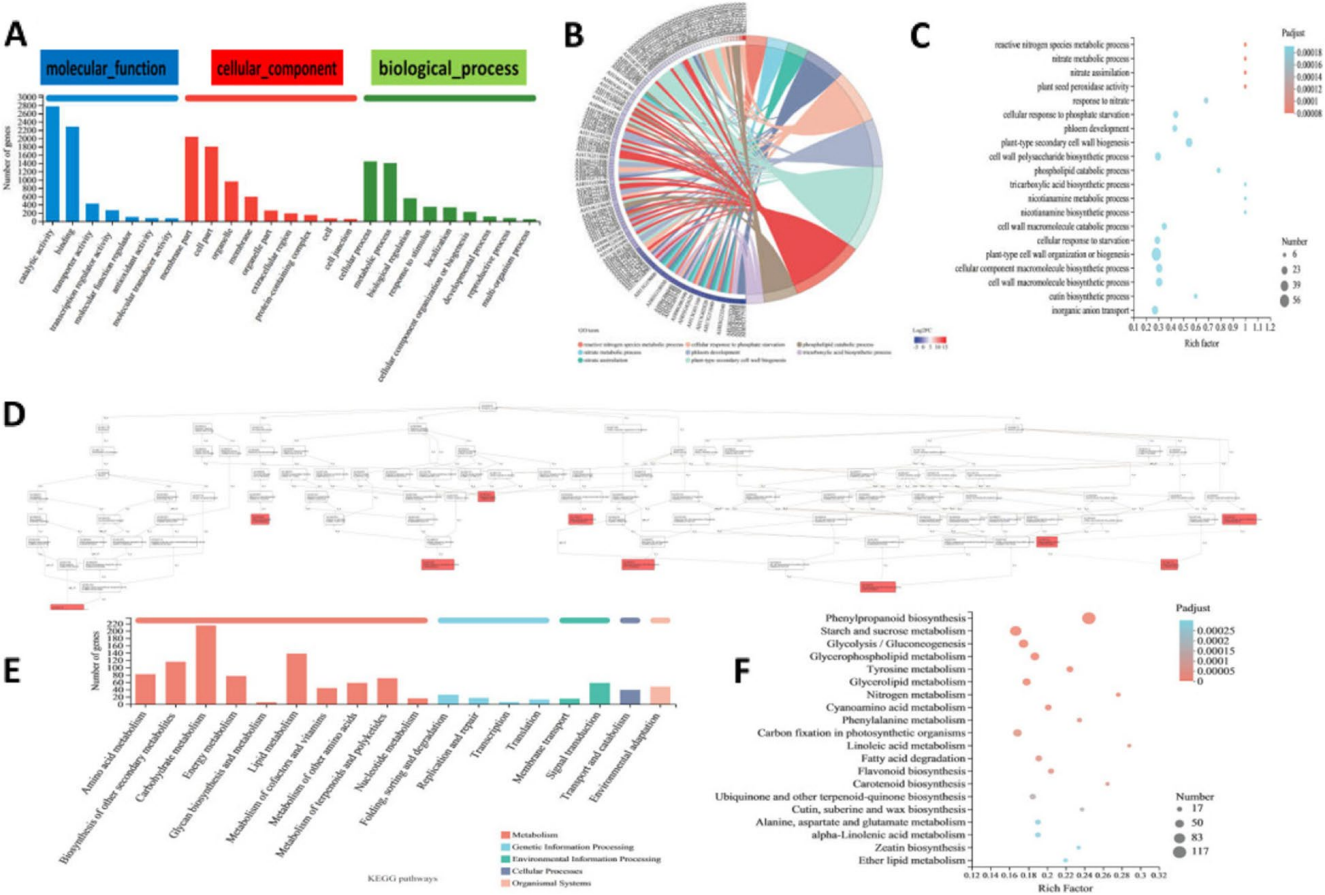
Analysis of transcriptome results. **A** GO annotation analysis. **B** and **C** GO enrichment analysis. **D** Directed acyclic graphs of GO annotation. **E** and **F** KEGG pathway annotation analysis and KEGG enrichment analysis

There were 6088 DEGs between –P treatment and + P treatment by using the criteria of false discovery rate (FDR) < 0.05 and |log_2_(fold change)|≥ 2. Compared with + P treatment, there were 3577 up-regulated and 2511 down-regulated DEGs under –P treatment (Supplemental Fig. S2; Supplemental Dataset S1). A total of 6088 DEGs were assigned both GO annotation and GO classification (Fig. 3A, B). Among those DEGs, in the "molecular function" category, 2772 and 2283 genes were in "binding" and "catalytic activity", respectively. In the "cellular component" category, the major genes were in "cell part" (1803) and "membrane part" (2040). Finally, in the "biological process" category, 1405 and 1444 were concentrated in "metabolic process" and "cellular process", respectively. Twenty GO terms were enriched in DEGs according to GO annotation analysis. A total of 56 genes were enriched in the top category of "plant-type cell wall organization of biogenesis" (Fig. 3C). The analysis of directed acyclic graph was conducted to focus on the GO structure (Fig. 3D). It is similar to GO analysis, three GO categories including "cell response to phosphate starvation", "peroxidase activity", "plant-type secondary cell wall biogenesis" were highlighted. The KEGG pathway analysis showed that functional annotation of DEGs was enriched into 18 subcategories (Fig. 3E). The most enriched KEGG orthology term was phenylpropanoid biosynthesis with 117 DEGs (Fig. 3F). Phenylpropane compounds played the important role in plant growth and stress response. The results suggested that genes of this pathway were closely related to P deficiency stress. This KEGG annotation provided valuable clues for investigating the genes and pathways involved in low P stress. Further joint analysis with metabolome was conducted and provided below in metabolome results.

**Fig. 4:**
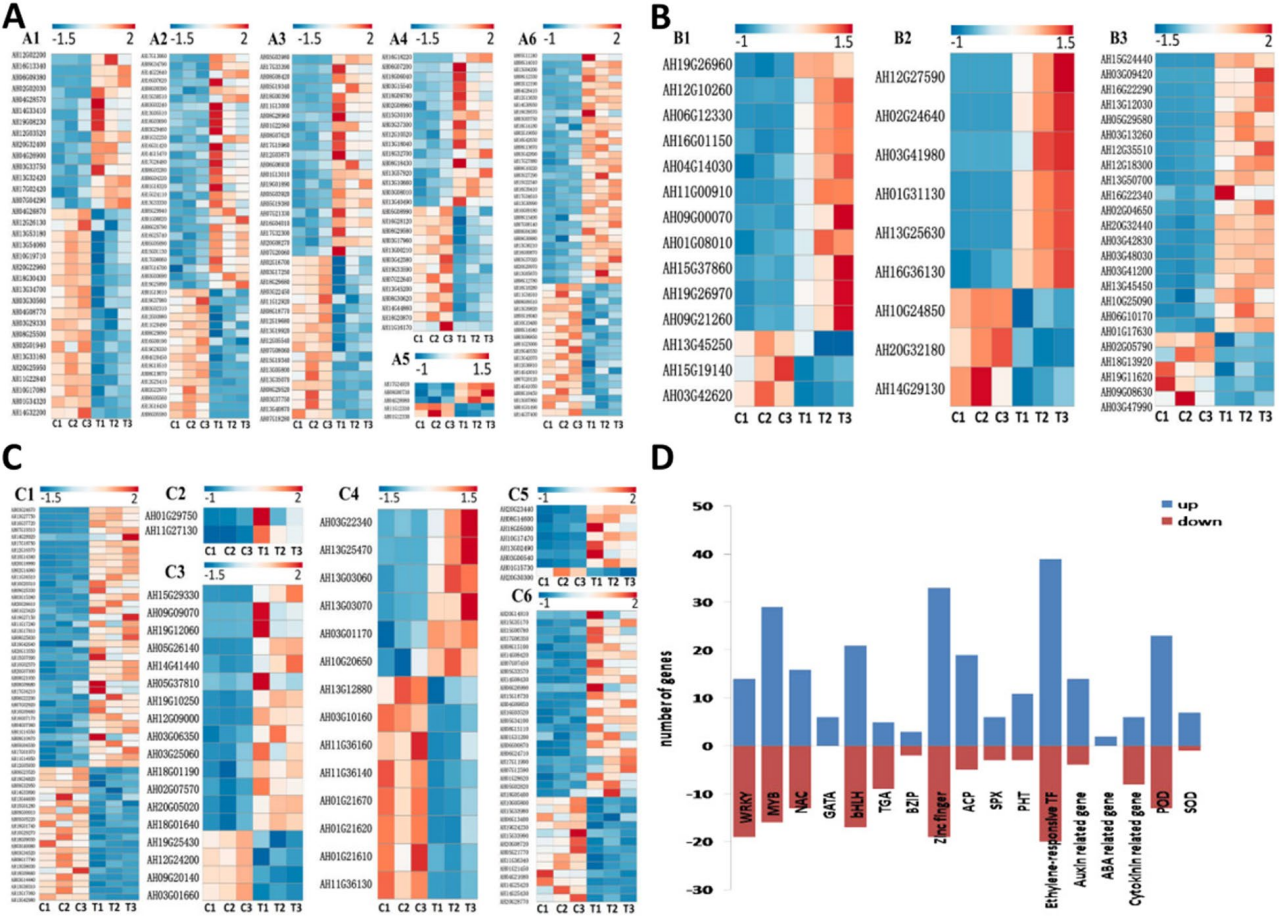
Transcriptional profiling of P deficiency related genes in the roots. **A** Six TF families: WRKY (A1), MYB (A2), bHLH (A3), NAC (A4), bZIP (A5), Zinc finger (A6) family genes. **B** DEGs encoding P uptake related genes: The DEGs of peanut *PHT* (B1), *SPX* (B2), and *ACP* (B3) genes in the roots under P deficiency. **C** DEGs encoding hormone metabolism related genes and antioxidant enzyme related genes. (C1) ethylene response transcription factors (*ERF*). (C2) *ABA* related genes. (C3) auxin response factor (*ARF*), small auxin upregulated RNA (*SAUR*), auxin binding protein genes (*ABP*), auxin induced protein genes. (C4) CTK*CTK* related genes. (C5) *SOD* genes. (C6) *POD* genes. **D** The number of up-regulated and down-regulated genes in 17 gene families under low p stress. C, control (+ P) group; T, treatment (–P) group

Many DEGs represented genes that were demonstrated to be related to responses to plant P deficiency stress in previous reports [29–31]. In particular, several transcription factor (TF) family genes, phosphate transporter genes, hormone metabolism related genes and antioxidant enzyme related genes that might be associated with P deficiency tolerance were detected (Supplemental Table S3). Under P deficiency stress, the induced expression of TFs, such as the WRKY family (Fig. 4A1), MYB family (Fig. 4A2), bHLH family, (Fig. 4A3) NAC family (Fig. 4A4), bZIP family (Fig. 4A5), and zinc finger family (Fig. 4A6) were identified. In this study, the results suggested these TFs played the crucial role in the transcriptional regulation of downstream genes related to P deficiency. In plants, the transport and absorption of P were conducted by phosphate transporters including the PHT family (Fig. 4B1) and SPX family (Fig. 4B2). PHT and proteins containing the SPX domain played the key role in maintaining P homeostasis and fine adjustment of P transport signaling [32–34]. In this study, *PHT* genes and *SPX* family genes were also found to be related under P deficiency stress. A total of 11 *PHT* genes and 6 *SPX* genes were up-regulated under –P treatment. In addition, one *PHR1 *(AH04G19770) has been identified up-regulated in –P group, which could positively regulate downstream *PHT* genes by binding the P1BS domain in promoter region. ACP played an important role in root P absorption. The ACP activity was remarkably higher under –P treatment than that under + P treatment, and several *ACP* genes were also significantly up-regulated under –P treatment. There is a strong positive correlation between the higher expression level of *ACP* genes and the increased activity of ACP (Fig. 4B3). DEGs also included the genes encoding hormone metabolism related genes and antioxidant enzyme related genes. The *ERF* (Fig. 4C1), *CAR* (Fig. 4C2), *ARF*, *SAUR*, *ABP* (Fig. 4C3), *CTK* related genes (Fig. 4C4) and *SOD* genes (Fig. 4C5), *POD* genes (Fig. 4C6) were also noted. Many *ERF* genes could be induced by abiotic stress including stress related hormone ETH and ABA. And ERF proteins conferred tolerance by binding to the promoter regions and activating the transcription of responsive genes. ERFs were involved in the regulation of ethylene dependent transcription which positively or negatively regulated the expression level of downstream ethylene inducible gene [35]. Under –P treatment, the content of ethylene was significantly decreased which was likely associated with the different expression of 39 *ERF* genes up-regulation and 20 *ERF* genes down-regulation. Two *CAR* genes which belonging to a plant specific gene family, positively regulated ABA sensitivity [36], and were all up-regulated under –P treatment. Therefore, the increase ABA content under –P treatment might associate with the up-regulated expression of these genes. Auxin played an important role across whole lifespan of plant. *ARF*, *SAUR*, *ABP*, and auxin induced protein genes were all belonged to auxin response genes which could be triggered rapidly and precisely by temporal and spatial changes of auxin level. And the level changes of auxin response genes led to level changes of downstream response genes. The auxin signal transduction finely regulated in plant developmental processes. Under –P treatment, 18 auxin response genes were identified as DEGs. The content of IAA was detected, which showed decreasing significantly when P was in deficiency. Plants could accurately perceive small changes in auxin levels. Therefore, it was indicated that changes in auxin levels can be transformed into auxin response genes transcription signals, which involved in various processes of plant development. Both *CKX* (catalyze CTK degradation) genes and *LOG* (catalyze CTK biosynthesis) genes were identified differentially expressed between the two treatments. Under –P treatment, three *CKX* genes up-regulated and six were down-regulated. Three genes encoding *LOG* were up-regulated and two were down- regulated. The comprehensive effect contributed to the decrease of CTK activity in –P peanut roots. 7 of 8 genes encoding *SOD* genes and 23 of 36 *POD* genes were identified up-regulation under –P treatment which could well explain the results of the increased activities of SOD and POD. One metallothionein gene that exerted large effect on homeostasis and ROS scavenging was also identified in this study. In summary, the number of up-regulated and down-regulated genes in 17 gene families under low p stress was shown in Fig. 4D. Altogether 8 up-regulated genes and two down-regulated genes were confirmed to be highly consistent (*R*^2^ > 0.95) with the transcriptome results by qRT-PCR. The results of qRT-PCR proved the reliability of transcriptome sequencing (Supplemental Fig. S3).

### miRNAs and target genes analyses of peanut roots under P deficiency stress

The 6 libraries included 77.77 M raw reads in total with a mean of 12.9 M in each library. After filtering low quality reads and rRNA, tRNA, snoRNA, snRNA, and repbase sequences, we obtained total 67,627,933 miRNA clean reads (Supplemental Table S4). The lengths of miRNA in each sample ranged from 18 to 32 nucleotides (nt). Sequences between 21 to 24 accounts for the highest proportion (Supplemental Fig. S4). The distribution of top15 known miRNA expressed in each sample was shown in Supplemental Fig. S5.

DeSeq 2 software was used to analyses the significantly different expression level between two treatments. The average value of change only at default parameters p-adjust ≤ 0.05 and |log_2_FC|≥ 1 was accepted. In summary, we identified 6 miRNAs which showed significantly differential expression between + P and –P group. Among these, there were 2 up-regulated miRNAs including ahy-miR160-5p and ahy-miR3518 while 4 down-regulated miRNAs including ahy-miR408-5p, ahy-miR408-3p, ahy-miR398, and ahy-miR3515. To confirm the expression patterns results obtained by high-throughput sequencing, 5 miRNAs and 5 target genes were analyzed by qRT-PCR (Supplemental Fig. S6). The qRT-PCR primers were designed according to the sequences of miRNAs and target genes, respectively (Supplemental Table S5). Further analysis of qRT-PCR validated consistent expression patterns with the high-throughput sequencing.

**Fig. 5:**
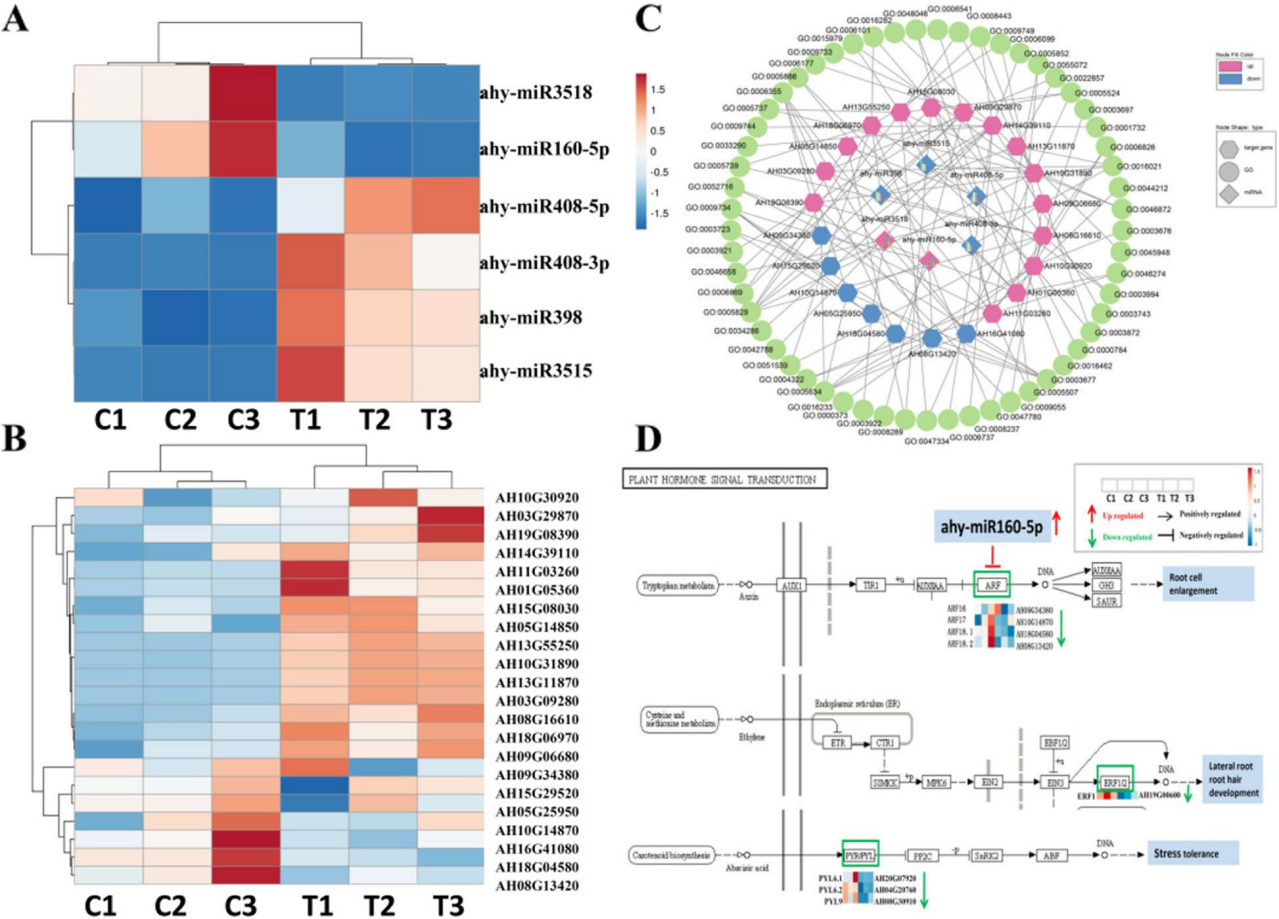
Different expressed miRNA analysis. **A** Heat map of six significant changed miRNAs. **B** Transcriptional profiling of target genes. **C** Significant different expressed miRNAs and its target genes. **D** miRNAs and its target genes related to plant hormone signal transduction

**Fig. 6:**
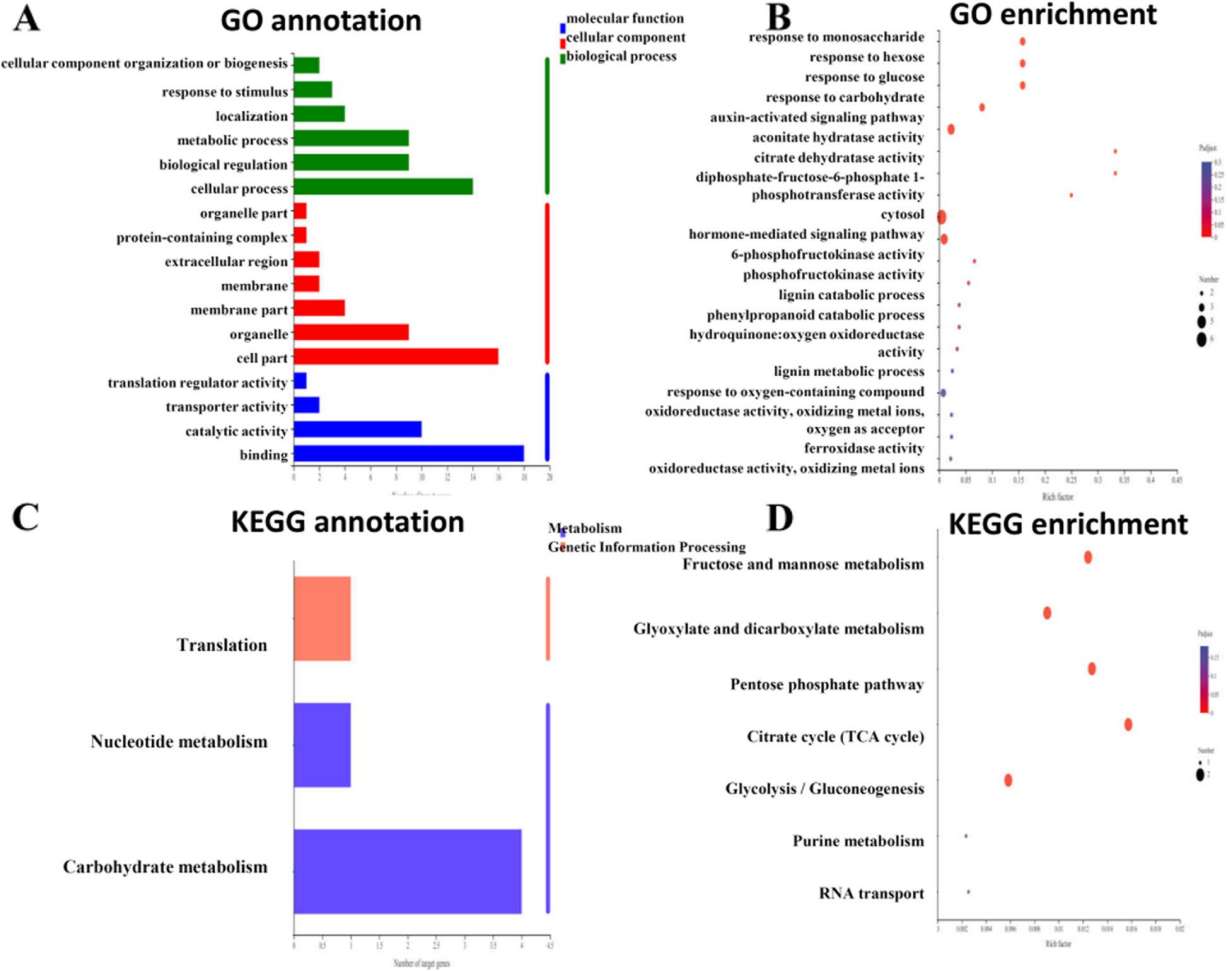
GO annotation and KEGG pathway analysis of target genes. **A** and **B** GO annotation and enrichment analysis. **C** and **D** KEGG pathway annotation and enrichment analysis

To analyze the functions of the identified miRNAs responding to P deficiency stress in peanut roots, we predicted the target genes by psRobot (http://omicslab.genetics.ac.cn/psRobot/). Comprehensive analysis of miRNAs and target genes can benefit for identification the regulatory function and miRNA-mRNA modules. A total of 6 P deficiency responding miRNA sequences were aligned to the peanut genome. Sequence complementarity between target mRNA and mature miRNA was screening criteria [37]. A total of 69 predicted target genes were obtained and the expression pattern of each target genes was subsequently analyzed in transcriptome database. After discarding the same expression pattern with corresponding miRNA and uncharacterized proteins, a total of 22 target genes for 6 miRNAs were found with higher or lower expression levels in P deficiency treatment (Fig. 5A, B; Table 1). The regulation network of miRNA-mRNA targets was conducted (Fig. 5C). To better understand the function of miRNAs, 22 predicted target genes according to transcriptome data were analyzed. Notably, 4 *ARF* genes have been found as the target genes of ahy-miR160-5p. The expression level of ahy-miR160-5p was significantly up-regulated while the 4 target genes were significantly down-regulated under –P treatment. The function of ARF family is responsive to auxin and regulating downstream auxin response genes by binding to promoters at *AuxREs*. It implies that ahy-miR160-5p involved in P deficiency stress by regulation of peanut auxin signal transduction pathway. Moreover, we also made integrated analysis of ETH and ABA signal finding that one down-regulated *ERF1* gene which was in the ETH signal transduction pathway and three down-regulated ABA receptor *PYL* genes in the ABA signal transduction pathway (Fig. 5D). Two glycerol-3-phosphate transporter 1 genes and two aconitate hydratase 3 genes were identified as the target genes of ahy-miR398. *PFP1*, the target gene of ahy-miR3515 is involved in regulation of starch metabolism. Transcriptome analysis revealed the expression level of *PFP1α* significant up-regulated in peanut –P stress. An MYB transcription factor *KAN4* has also been identified as the target gene of ahy-miR3518 which is involved in the regulation of flavonoid synthesis in *Brassica juncea* [38]*.* The expression level of two *YL3* genes targeted by ahy-miR408-5p were up-regulated in –P group. Generally, YL3 protein is a transfer protein for lipid and the gene was up-regulated during senescence in *Arabidopsis* [39]. Two laccase-3 genes is also identified as target gene of ahy-miR408-3p. Plant laccases has been reported involved in the lignin biosynthesis and the response to abiotic stress. The results indicated that significantly expression level change of miRNA and its target genes play vital and diverse roles in transcriptional level under –P stress. To gain insight of target genes function, GO categories were performed. Six classed of biological processes category were identified with "cellular process" the most frequent including 14 genes. In cellular component category, 16 target genes were enrichment of "cell part". In molecular function category, The targets were remarkably enriched in "binding" with 18 genes (Fig. 6A, B; Table 1). In addition, KEGG pathway enrichment analyses revealed that 22 target genes were enriched in 5 pathways significantly including "fructose and mannose metabolism", "citrate cycle (TCA cycle)", "pentose phosphate pathway", "glycolysis/gluconeoge" and "glyoxylate and dicarboxyl" (Fig. 6C and D). The results highlighted the miRNA-mediated regulation under P deficiency stress via involving in post-translational modulation.

**Table 1:** Predicted targets genes of peanut miRNAs in response to P deficiency

**miRNA **	**Target gene ID**	**Paired alignment**	**Target gene**	**Function annotation**	**GO annotation**
ahy-miR160-5p	AH18G04580		Auxin response factor 18	GO:0006355 GO:0009734 GO:0005634 GO:0003677	BP: regulation of transcription, DNA-templated BP: auxin-activated signaling pathway CC: nucleus MF: DNA binding
ahy-miR160-5p	AH09G34380		Auxin response factor 16	GO:0006355 GO:0009734 GO:0005634 GO:0003677	BP: regulation of transcription, DNA-templated BP: auxin-activated signaling pathway CC: nucleus MF: DNA binding
ahy-miR160-5p	AH10G14870		Auxin response factor 17	GO:0006355 GO:0009734 GO:0005634 GO:0003677	BP: regulation of transcription, DNA-templated BP: auxin-activated signaling pathway CC: nucleus MF: DNA binding
ahy-miR160-5p	AH08G13420		Auxin response factor 18	GO:0006355 GO:0009734 GO:0005634 GO:0003677	BP: regulation of transcription, DNA-templated BP: auxin-activated signaling pathway CC: nucleus MF: DNA binding
ahy-miR3515	AH10G30920		CST complex subunit STN1	GO:0016233 GO:0005634 GO:0000784 GO:0003676 GO:0003697	BP: telomere capping CC: nucleus CC: nuclear chromosome, telomeric region MF: nucleic acid binding MF: single-stranded DNA binding
ahy-miR3515	AH10G31890		Pyrophosphate--fructose 6-phosphate 1-phosphotransferase subunit alpha	GO:0009749 GO:0015979 GO:0005737 GO:0005829 GO:0047334 GO:0003872 GO:0046872 GO:0005524 GO:0008443	BP: response to glucose BP: photosynthesis CC: cytoplasm CC: cytosol MF: diphosphate-fructose-6-phosphate 1-phosphotransferase activity MF: 6-phosphofructokinase activity MF: metal ion binding MF: ATP binding MF: phosphofructokinase activity
ahy-miR3515	AH14G39110		Eukaryotic translation initiation factor 3 subunit H	GO:0009733 GO:0009744 GO:0034286 GO:0009737 GO:0009749 GO:0045948 GO:0001732 GO:0005852 GO:0016282 GO:0005829 GO:0033290 GO:0042788 GO:0008237 GO:0003743	BP: response to auxin BP: response to sucrose BP: response to maltose BP: response to abscisic acid BP: response to glucoseBP: positive regulation of translational initiation BP: formation of cytoplasmic translation initiation complex CC: eukaryotic translation initiation factor 3 complex CC: eukaryotic 43S preinitiation complex CC: cytosol CC: eukaryotic 48S preinitiation complex CC: polysomal ribosome MF: metallopeptidase activity MF: translation initiation factor activity
ahy-miR3515	AH13G55250		Pyrophosphate--fructose 6-phosphate 1-phosphotransferase subunit alpha	GO:0009749 GO:0015979 GO:0005737 GO:0005829 GO:0047334 GO:0003872 GO:0046872 GO:0005524 GO:0008443	BP: response to glucose BP: photosynthesis CC: cytoplasm CC: cytosol MF: diphosphate-fructose-6-phosphate 1-phosphotransferase activity MF: 6-phosphofructokinase activity MF: metal ion binding MF: ATP binding MF: phosphofructokinase activity
ahy-miR3518	AH15G29520		CRS2-associated factor 1, chloroplastic	GO:0003723	MF: RNA binding
ahy-miR3518	AH05G25950		CRS2-associated factor 1, chloroplastic	GO:0000373 GO:0003723	BP: Group II intron splicing MF: RNA binding
ahy-miR3518	AH16G41080		Probable transcription factor KAN4	GO:0006355 GO:0005634 GO:0044212 GO:0003677	BP: regulation of transcription, DNA-templated CC: nucleus MF: transcription regulatory region DNA binding MF: DNA binding
ahy-miR398	AH08G16610		Aconitate hydratase 3, mitochondrial	GO:0006099 GO:0006101 GO:0005829 GO:0005739 GO:0051539 GO:0047780 GO:0003994	BP: tricarboxylic acid cycle BP: citrate metabolic process CC: cytosol CC: mitochondrion MF: 4 iron, 4 sulfur cluster binding MF: citrate dehydratase activity MF: aconitate hydratase activity
ahy-miR398	AH13G11870		Putative glycerol-3-phosphate transporter 1	GO:0016021 GO:0022857	CC: integral component of membrane MF: transmembrane transporter activity
ahy-miR398	AH03G29870		GMP synthase [glutamine-hydrolyzing]	GO:0006541 GO:0006177 GO:0005829 GO:0003921 GO:0003922 GO:0016462 GO:0005524	BP: glutamine metabolic process BP: GMP biosynthetic process CC: cytosol MF: GMP synthase activity MF: GMP synthas MF: pyrophosphatase activity MF: ATP binding
ahy-miR398	AH18G06970		Aconitate hydratase 3, mitochondrial	GO:0006099 GO:0006101 GO:0005829 GO:0005739 GO:0051539 GO:0047780 GO:0003994	BP: tricarboxylic acid cycle BP: citrate metabolic process CC: cytosol CC: mitochondrion MF: 4 iron, 4 sulfur cluster binding MF: citrate dehydratase activity MF: aconitate hydratase activity
ahy-miR398	AH03G09280		Putative glycerol-3-phosphate transporter 1	GO:0016021 GO:0022857	CC: integral component of membrane MF: transmembrane transporter activity
ahy-miR408-3p	AH19G08390		Blue copper protein	GO:0046658 GO:0016021 GO:0009055	CC: anchored component of plasma membrane CC: integral component of membrane MF: electron carrier activity
ahy-miR408-3p	AH11G03260		Laccase-3	GO:0046274 GO:0006826 GO:0055072 GO:0048046 GO:0005886 GO:0052716 GO:0004322 GO:0005507	BP: lignin catabolic process BP: iron ion transport BP: iron ion homeostasis CC: apoplast CC: plasma membrane MF: hydroquinon MF: ferroxidase activity MF: copper ion binding
ahy-miR408-3p	AH01G05360		Laccase-3	GO:0046274 GO:0006826 GO:0055072 GO:0048046 GO:0005886 GO:0052716 GO:0004322 GO:0005507	BP: lignin catabolic process BP: iron ion transport BP: iron ion homeostasis CC: apoplast CC: plasma membrane MF: hydroquinon MF: ferroxidase activity MF: copper ion binding
ahy-miR408-3p	AH09G06680		Blue copper protein	GO:0046658 GO:0016021 GO:0009055	CC: anchored component of plasma membrane CC: integral component of membrane MF: electron carrier activity
ahy-miR408-5p	AH15G08030		Protein YLS3	GO:0006869 GO:0008289	BP: lipid transport MF: lipid binding
ahy-miR408-5p	AH05G14850		Protein YLS3	GO:0006869 GO:0008289	BP: lipid transport MF: lipid binding

In addition, to further understand of miRNA regulatory network, 2000 bp upstream of 6 miRNA genes were scanned for TSSs and *cis*-elements (Table 2). Totally, 11 putative *cis*-elements were identified for 6 miRNAs in peanut responsive to P deficiency stress. For instance, one w-box element was found in ahy-miR408 promoter which is the WRKY TFBS) while four bZIP transcription factor binding sites were identified in promoters of ahy-miR398, ahy-miR408 and ahy-miR3515. Interestingly, one ERRE element was found in ahy-miR3518 promoter which was the binding site for EREBP, RAP2.6 and ERF1 transcription factors. EREBP TF family members might play an crucial role in response to ABA signal pathway in stress [40]. RAP2.6 belongs to ERF proteins which is also responsive to ABA and different environment stress such as salt and cold [41]. One L3 element was found in ahy-miR160-5p promoter which can be bound by Dof11 transcription factors. It is reported that Dof11 was a sugar transporter and can increase resistance to *Rhizoctonia solani* which causes sheath blight disease in rice [42].

**Table 2:** Putative transcription start sites (TSSs) and transcription factor binding sites (TFBSs) in 2000 bp promoter of P deficiency responsive miRNA genes in peanut

**miRNA**	**miRNA sequence**	**regulation**	**pre-miRNA**	**pre-miRNA sequence**	**Chromosome**	**TSSs prediction**	**TFBSs prediction**	**TF prediction**
ahy-miR408-5p (21bp)	CUGGGAACAGG CAGAGCAUGA	down	ahy-MIR408 (122bp)	GAAGAAGAGAUGACAAAGAACUG GGAACAGGCAGAGCAUGAAUGGA ACUAUCAAUAGACACAUUUUGUUC AUUGACGCUCAUGCACUGCCUCUU CCCUGGCUCUCUCUUUCUUUUUCC UUCU	Chr06	-1788 -1342 -1005 -700- 363 -62	ACGT-containing element (ACE1) 1208 ACACGTAG 1215 W box 1145 AATTGGTCAATATG 1158	HY5 (bZIP transcription factor) WRKY1
ahy-miR408-3p (21bp)	AUGCACUGCCU CUUCCCUGGC	down	ahy-MIR408 (122bp)	GAAGAAGAGAUGACAAAGAACUG GGAACAGGCAGAGCAUGAAUGGA ACUAUCAAUAGACACAUUUUGUUC AUUGACGCUCAUGCACUGCCUCUU CCCUGGCUCUCUCUUUCUUUUUCC UUCU	Chr06	-	-	-
ahy-miR398 (20bp)	UGUGUUCUCAG GUCACCCCU	down	ahy-MIR398 (153bp)	UAUCUCAGAGGAGUGAACCUGAGA ACACAAAGUAAAUUGUUUCGGAG UUUGGAAUGCCAUAAUCACAUGCA UAAUGCAUUUAUGCUUAUGCUUG UAUAUGGUACAUAUUUUCGAAUU UUAAUUUACCUUGUGUUCUCAGGU CACCCCUUUGAG	Chr19	-1669 -1331 -798 -490 -177	hor1 box 1861 GTGAGTCAT 1869	BLZ1 (bZIP transcription factor)
ahy-miR3515 (21bp)	AAUGUAGAAAA UGAACGGUAU	down	ahy-MIR3515 (174bp)	GGAGGAGAAUCAAUAAUGUUAUU UCUACAUUUCAUUUUCUAUACUCA UUCUCUGCACCUUCCUUUUAUGUA UGAUAAAGGAAAAAAUAUAAAUU UUUCUAUUAUUUUGUCAUAAAAG UGAGGUGUAGAGUAAAAAUGUAG AAAAUGAACGGUAUAAAUAACAU UGAUGUUGAGA	Chr15	-1616 -1334 -745 -440 -127	RY/G box 1673 CAtGTG 1678 -- 5-- 1684 CATGCA 1689 ZDRE 1756 ATGTCGACAT 1765 CG 659 aAACCGTCGATa 670	ABI3 bZIP19; bZIP23 WLIM2
ahy-miR160-5p (21bp)	UGCCUGGCUCC CUGAAUGCCA	up	ahy-MIR160 (126bp)	AAAUAUUCGAAUUAUGUGUCUGCC UGGCUCCCUGAAUGCCAUGUAAGU UAGUUUGUUAAGAAAAGAUUAAC AAACGUCUUAUAUGGCAUGAAGG GAGUCACGCAGGCGAUUCAUUUUG AAACAUAU	Chr14	-1682 -1348 -1031 -577 -181	AG2 1710 TcCCCAATGTcTT 1722 L3 1495 tAAAAGtgTAAAGAGA 1510	LFU; Dof11
ahy-miR3518 (21bp)	UGACCUUUGGG GAUAUUCGUG	up	ahy-MIR3518 (160bp)	GCUAAGCGACAAUAAUUUGAGAG AUAAUAUGUAACGAGUAUCACCAA AUGUCACUCUUUUAGAUUUGUCUU AUUAGUUAAGGAGUUGCAACUUA UGAAGUAAAGGAUAAAUUAAAAG AGUGACCUUUGGGGAUAUUCGUG GCUCAUUACUUCUCAAAUUA	Chr09	-1557 -1249 -861 -347	ERRE 850 AGCCGCC 856 878 AGCCGCC 884 B1 1367 GTTGACATGG 1376 EBP BS 850 AGCCGCCAT 858 878 AGCCGCCGT 886	EREBP; RAP2.6; ERF1 Opaque-2 EBP

### Metabolites of peanut roots in response to P deficiency

A total of 439 DAMs showed obviously differences in two experimental treatments (Supplemental Dataset S2). OPLS-DA analysis showed that the model was reliable and VIP analysis could reflect contributions of DAMs between treatments (Supplemental Fig. S7A). The top two clusters characterized by 81 down-regulated metabolites including lysoPC(18:0), lysoPC(15:0), lysoPC(18:1(9Z)), lysoPC(18:2(9Z,12Z)), lysoPE(0:0/20:4(5Z,8Z,11Z,14Z)) and 120 up-regulated metabolites including L-tryptophan, L-arginine, phenylalanyl-Lysine, 9(S)-hpOTrE (9(S)-HPOT), indicated the involvement of these metabolites in coping with low P stress. (Supplemental Fig. S7B). Among 439 DAMs, 334 metabolites were significantly accumulated while 105 metabolites were remarkably decreased in low P group (Supplemental Fig. S8A). The PCA for all samples showed that replicates of each group well clustered together. PC1 (horizontal axis) and PC2 (vertical axis) can explain 45% and 8.83% of the variance, respectively (Supplemental Fig. S8B). Based on the variation of metabolite composition and abundance of each sample, biological replicates in the same treatment show well correlation (Supplemental Fig. S8C). The results highlighted the metabolites changes occurred after –P treatment. According to the expression pattern of metabolites, metabolite correlation analysis has been performed. The same patterns of metabolites has been clustered by *k*-means method (Supplemental Fig. S8D). The result showed that under low P stress peanut exhibited strong phenylalanine metabolism, which was consistent with transcriptome analysis. In addition, a significant increase of several amino acid were detected. However, phosphatidylcholine (lecithin) decreased significantly. Further, metabolites were analyzed by KEGG compounds classification (Supplemental Fig. S9A) and HMDB 4.0 database (Supplemental Fig. S9B). To identified the metabolic pathway of DAMs involved in, KEGG pathway classification and enrichment analyses were conducted (Supplemental Fig. S9C, S9D). The results showed that P deficiency generally positively regulated the abundance of several amino acids in N metabolism, JA in α-linolenic acid metabolism, levan in sucrose metabolism, and some products in phenylpropanoid biosynthesis pathway. On the contrary, lysoPC (LPC) involved in glycerolipid metabolism were down-regulated significantly under –P stress.

**Fig. 7:**
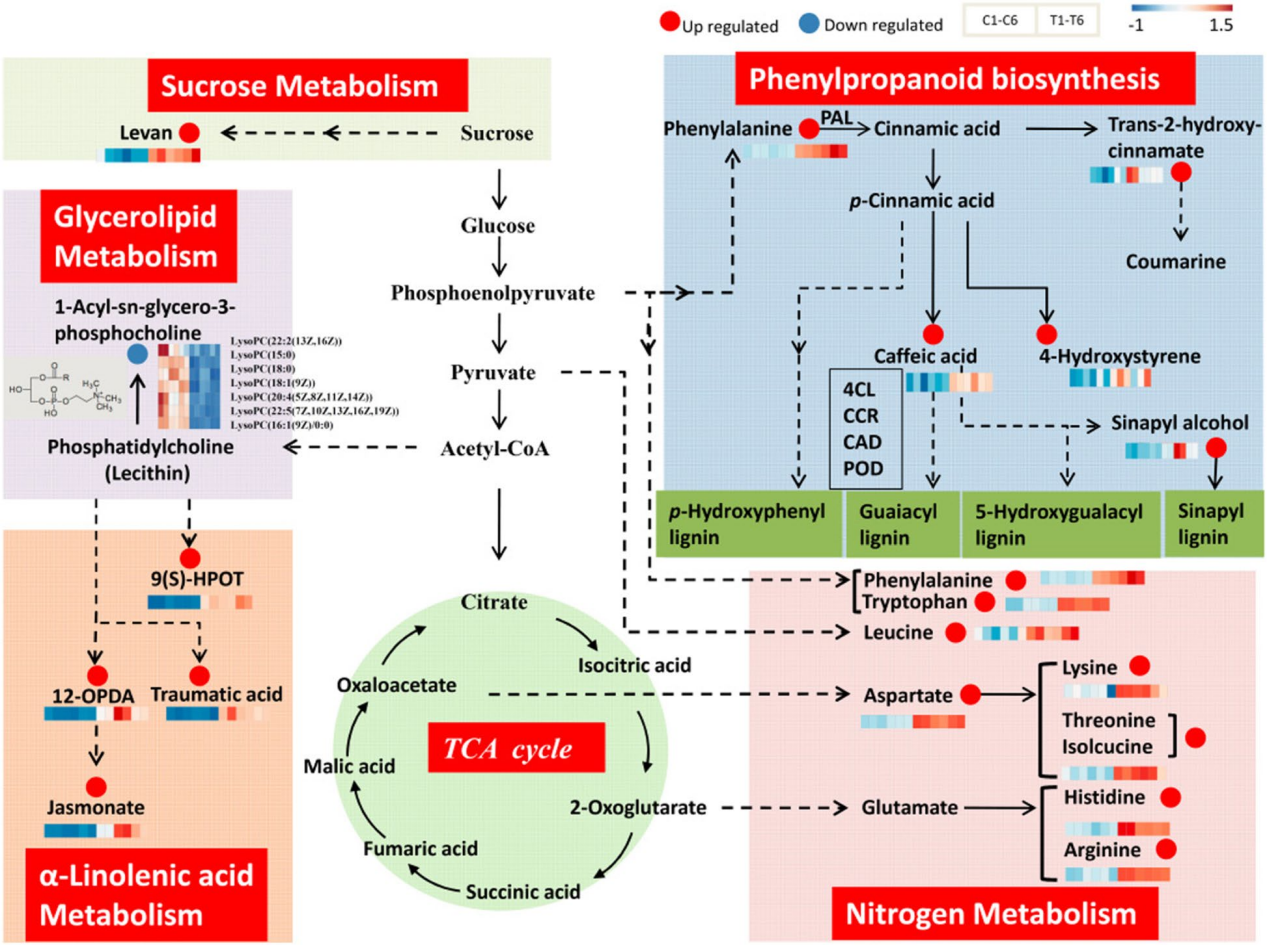
Candidate DAMs and metabolic pathway related to the low P responding. PAL, phenylalanine lyase; 4CL, 4-coumadin CoA ligase; CCR, cinnamoyl-CoA reductase; CAD, cinnamyl alcohol dehydrogenase; POD, peroxidase

The DAMs involved in the nitrogen metabolism were visualized in Fig. 7. Low P stress generally positively regulated the abundance of amino acids and its intermediates. Two aromatic amino acids (phenylalanine and tryptophan), two branched-chain amino acid (BCAA) (leucine and isoleucine), and three alkaline amino acid (lysine, histidine and arginine) were significantly accumulated under low P stress. Furthermore, the increase of phenylalanine and tryptophan pointed to a function of P deficiency tolerance and phenylpropanoid biosynthesis pathway which was significantly increased. Phenylalanine, one product of plant shikimate pathway, was the entry of the phenylpropanoid biosynthesis. PAL catalyzed the phenylalanine into trans-2-hydroxy-cinnamate and direct carbon flow enter into the biosynthesis of multiple metabolite and derivative in phenylpropanoid metabolism pathway. The accumulation of phenylalanine, trans-2-hydroxy-cinnamate, caffeic acid, 4-hydroxystyrene, and sinapyl alcohol were up-regulated. Joint analyses with transcriptome, 4 enzymes which played important roles in the substeps of phenylpropanoid metabolism showed obviously increasing expression. The accumulations of PAL, 4CL, CCR, CAD were significantly up-regulated. The enzyme activity of POD was also up-regulated by above enzyme activity test although 23 *POD* genes were up-regulated and 13 genes were down-regulated in transcriptome. PAL, 4CL, CCR, CAD, and POD were vital for the feeding into the biosynthesis of guaiacyl (G) lignin, syringyl (S) lignin, 5-hydroxy-guaiacyl (H) lignin, and p-hydroxy-phenyl (P) lignin. The significance and enrichment up-regulation in this pathway indicated that under –P treatment, the increased phenylalanine may direct the entire biosynthesis of phenylpropanoid towards to the production of lignin to increase the tolerance. In linolenic acid metabolism, intermediate products of 9(S)-HPOT, 12-OPDA (12-oxo-phytodienoic acid), traumatic acid and JA were all up-regulated. The phytohormone JA derived from α-linolenic acid was a critical signal involved in both abiotic and biotic stress responses as well as multiple developmental processes [43, 44]. 12-OPDA which was metabolic precursor of JA and could generate JA by undergoing a several rounds of oxidation reaction. Both 12-OPDA and its metabolite JA directly or indirectly activated downstream genes and caused defense response. The results showed that JA can be induced by low P stress. Levan, as an important osmotic regulator, was produced from sucrose and closely related to resistance of plants under stress. In this study, the results was consistent with previous studies that the accumulation of levan could be induced by nutritional stress [45]. In addition, seven kinds of LPC were identified down-regulated in glycerophospholipid metabolism pathway remarkably. LPC is a derivative of phosphatidylcholine (PC or lecithin) which was the major component of cell membrane.

## Discussion

Peanut is a P-loving crop, which plays an very important role in edible oil supply. P is one of the most essential and easily lacking nutrient for peanut [46]. However, many soils suitable for peanut planting always lack of P nutrition both in China and many other countries and areas of the world [4, 5]. Through a long evolutionary process, plants have evolved complex strategies to deal with P deficiency [47]. Unavailability of P can cause adaptive changes in peanut phenotypes, physiology and microscopic, which are ultimately reflected in plant growth retardation and yield reduction [6, 7].

### Peanut growth and development inhibition under P deficiency stress

To assess the phenotypic alterations of plants under the two treatments, the present study detected peanut growth and development indexes of aboveground parts and roots. In agreement with previous reports [48–50], the results showed that peanut under 60 days long term P deficiency stress at seedling stage are dwarfed. The peanut root system is the main organ for P absorption. These studies demonstrated that the morphology and structure of roots were the most pronounced changes under P deficiency stress which exerted a distinct effect on root morphological variation [51]. Meanwhile, the P content in the growth environment directly affected the P content in the roots. For the sake of photosynthesis in the leaves, the plant could adjust the limited P to give priority to the leaves. Therefore, under P deficiency stress, the total P content in the roots showed the greatest decrease. In addition to increasing P absorption by adjusting root morphology, roots also secreted ACP to promote the hydrolysis of soil organic P phospholipid bonds, releasing absorbable H_2_PO_4_^–^ and HPO_4_^2–^, ultimately achieving P absorption. When the plant lacked of P during the growth process, in order to reduce the level of P deficiency stress, the plant itself could synthesize more intracellular ACP to decompose the phospholipids in the body for the needs. As the stress intensifies and time passes, the plant synthesized a large amount of secreted ACP into the environment to turn the organic P into inorganic P for direct absorption and utilization [14, 52, 53]. Hence, we checked the changes in ACP enzyme activities between two treatments. It was difficult to measure the activity of ACP secreted into the hydroponic culture in this study, but the results of ACP activity in roots showed that the ACP activity under –P treatment was significantly higher than the ACP activity under + P treatment. The transcriptome data also indicated that several *ACP* genes were also significantly up-regulated under –P treatment. The results showed that peanut plants would indeed produce more ACP to adapt to P deficiency stress. Extremely no P application also hampered the photosynthetic system by reducing leaf growth and photosynthetic function [18, 54]. Long-term in P deficiency could limit the formation of photosynthetic pigments and the ability to utilize sunlight with reduction in the A and Gs values in different plant species [55]. Our results were in good agreement with the above literature. Severe P deficiency affected the quality and quantity of leaf cells as phenotypic total leaf area reduction and photosynthetic capacity [56]. The external form and internal structure of plants are closely related to the external environment. The root, a key organ for nutrient absorption, can adapt to P deficiency stress by changing morphology and structure. In addition, P deficiency leads to root development and physiological dysfunction. By detecting root anatomical structure in two treatments, it is shown that the effect of no P stress on root microstructure was significant. When P supply was sufficient, root growth and development were normal, and the roots could fully absorb and utilize P. Under –P stress, the morphology of roots changed to adapt to the environment. The taproot phenotype was weak and fragile, while the anatomic structure was abnormal. Then the cortex was seriously damaged, with some parenchymal cells that were irregular or even broken. The cell arrangement in the vascular bundle also became loose.

P deficiency is seriously destructive to peanut growth. The effects of nutrient deficiency will accumulate to a level causing the imbalance of ROS [15, 57]. ROS is reported to be cytotoxic, mutagenic and destructive to cell organelles, including DNA, lipids, proteins and carbohydrates. When P is deficient, ROS might accumulates in plant cells. However, the amount of ROS needs to be maintained at a safe level for survival. Internal antioxidant enzymes such as SOD, POD, and CAT can protect plants against ROS damage. The induction of the antioxidant system can improve the tolerance of plants to P deficiency stress [58, 59]. In this study, CAT and SOD activities increased significantly in roots under P deficiency stress compared to the POD and SOD activities under P sufficiency condition. CAT, SOD and POD were also higher in leaves but with no significance. That meant the antioxidant enzyme system in roots played a crucial physiological function, as a protectant to scavenge ROS [17]. The activities of GS, IAA and CTK are also related closely to the P deficiency stress. GS is a key enzyme controlling nitrogen utilization and is an important physiological index for measuring the level of plant N assimilation. The content of GS may be regulated by IAA concentration. The 5'-terminus of the GS gene promoter containing a *cis*-acting element can specifically bind to IAA [60]. IAA regulates the elongation and division of plant cells and the growth of taproots and lateral roots. CTK in plants is synthesized mainly at the root tip. CTK has a synergistic effect with plant IAA and can promote the differentiation rate of cells to promote plant growth and development. Compared to + P treatment, the change in ETH activity was similar to that of GS and IAA but with a remarkable reduction in roots (*p* < 0.01) and a notable increase in leaves (*p* < 0.01) under –P treatment. ETH is widely involved in the regulation of plant growth and development and various stress responses. ETH can positively regulate the development of root hair [61]. However, ABA can inhibit root hair growth [62]. The results of ABA activity change were the opposite of the results of ETH activity change in both roots and leaves, with significance. ABA can be regulated by abiotic stress. Under P deficiency stress, its synthesis was increased whereas catabolism was decreased, resulting in an increase in the total amount [63]. NR is the rate-limiting enzyme of N assimilation in higher plants and can directly regulate N metabolism. The activity of NR was promoted by ETH and CTK but inhibited by ABA [64]. These results were in agreement with our observations in P-deficient peanuts. The treated peanut plants showed lower levels of NR, which may ultimately lead to an induced accumulation of biomass. As a result, the authors speculated that P in deficiency led to the decreased activities of GS, IAA, ETH, NR, and CTK and increased activity of ABA. The joint regulation of these enzymes caused the significant phenotypic differences in plant roots.

### Transcriptome analysis of peanut roots under P deficiency stress

P deficiency is one of the most serious abiotic stresses of the peanut. The most obvious effect of P deficiency is the inhibition of root growth. In our study, to explore candidate genes in response to P deficiency stress in peanut, transcriptome analysis was performed under either –P or + P conditions. The results showed that several TFs, phosphate transporter genes, hormone response-related genes, and antioxidant enzyme genes were up-regulated in roots under P deficiency stress. These genes were involved in nutritional metabolism, stress response, and oxidoreductase activity. TFs were proven to be important transcriptional regulators in response to stress by regulating downstream stress response genes. Many genes of the WRKY TF family, MYB TF family and NAC TF family were reported to be related to the regulation of P absorption [65]. The results also revealed that the expression of *PHT* genes and *SPX* genes were increased. Most of the PHT family and SPX family were responsible for P absorption. Both of these families could be regulated by MYB TF family and WRKY TF family [66, 67]. Genes of those TF family was identified as P deficiency responsive genes by transcriptome analysis. Root ACP played an important role in P absorption. In this study, the expression levels of several *ACP* encoding genes were up-regulated and the content of ACP in roots was also significantly higher under –P treatment. DEGs also included *ERF*, *ABA* related genes, *ARF*, *SAUR*, *ABP*, auxin induced protein genes, *CTK* related genes and *SOD* genes, *POD* genes. Under –P treatment, the expression levels of genes encoding hormone metabolism related genes and antioxidant enzyme related genes were changed compared with that under + P treatment. The results suggested that P deficiency stress influenced hormone metabolism and overproduced cytotoxic ROS. It was indicated that hormone signal transduction and regulation played a crucial role under P in deficiency. Phenylpropanoid biosynthesis was identified by KEGG, which can lead to the accumulation of diverse phenolic compounds such as lignin under various abiotic stress (like nutrients deficiency, drought, wounding) to maintain the stability of plants [68, 69].

### miRNA analysis of peanut roots under P deficiency stress

Under –P stress, the development of peanut plant, especially roots, was inhibited remarkably including shortened root, abnormal growth. Regulation of P transport by miRNAs mediated post-transcription have been shown responsive to external P deficiency [70]. The high-throughput sequencing of miRNA survey identified 6 responsive miRNAs which would be involved in the response and regulation of P deficiency stress pathway in peanut. In present study, the expression level of ahy-miR160-5p was sufficiently up-regulated in P deficiency. Many previous studies reported that miR160 was responsible for auxin and ABA signalling pathway during stress conditions [71, 72]. The miR160 acted as a negative regulator in auxin signalling through degradation of a member of *ARF* genes. Four target genes of ahy-miR160-5p belonging to *ARF* gene family had been predicted (*ARF16*, *ARF17*, *ARF18.1*, *ARF18.2*). *ARF16* and *ARF17* had been reported played an important role in root developmental process [73, 74]. Transcription results of ahy-miR160-5p and its 4 target genes were increased or decreased under –P stress, respectively. The down-regulated expression level of 4 *ARF* genes may resulted by the accumulation of ahy-miR160-5p. It may cause the auxin response and root developmental process suppressed. On the contrary, repressed level of ahy-miR398 was detected. Many preliminary analysis suggested that miR398 was responsive to P deficiency in various species and played a crucial role in low P stress regulation [75, 76]. In addition, target genes of miRNA398 have been shown to be related with P homeostasis under P starvation in plant [77]. In this work, two putative *GlpT1* were predicted as the target genes of ahy-miR398, which were vital for P uptake by transporting inorganic phosphate into cytoplasm [75, 78]. Similarly, ahy-miR408 was shown repressed level in –P stress which was identified as PSR miRNA in multiple species [79] and the expression level is controlled by the availability of copper [80]. Two blue copper protein genes and two laccase-3 genes which encoding copper-containing proteins have been identified as the target gene of ahy-miR408. Laccase was always involved in biosynthesis of lignin which could be induced by limited nutrients such as P [81, 82]. In addition, Lignin can be synthesized begins with phenylalanine and tyrosine in phenylpropanoid biosynthesis pathway [83] which has been identified by transcriptome KEGG orthology analysis in this work. However, the mechanisms of P uptake were associated with the global miRNAs regulatory network which cause a series of physiological changes including not only P metabolism but also overall plant growth [84]. The P starvation induced changes of multiple TF genes express involving in diverse functions. Detailed characterizations of promoters in 6 ahy-miRNAs genes revealed that several binding sites for TFs responding to comprehensive functions existed. For instance, one ACE1 was identified in the promoter of ahy-MIR408 which was the *cis*-elements for *HY5* TFs. It is known that miR408 was an crucial component of HY5-SPL7 network which can mediate the plant to response to environment stress [85]. The results suggested that miRNAs could response to many stresses for containing diverse responsive *cis*-elements. Meanwhile, this is a well explanation for the regulation network of P deficiency was redundancy with other stress response.

### Metabolome analysis of peanut roots under P deficiency stress

Metabonomic has become an effective approach to evaluate the changes in multiple metabolites in post transcriptional regulation. In this study, to investigate the alternation of metabolites accumulation under P deficiency stress in peanut roots, we conducted the metabonomic analyses. The results demonstrated strong phenylpropanoid metabolic activity during P deficiency, which has also been identified in P deficiency trascriptome analysis. Phenylpropanoid metabolism which cascade from phenylalanine to various lignin formation always played an important role in response to environmental stress. A total of 5 increased metabolites were enriched in phenylpropanoid biosynthesis pathway. PAL, the gateway enzyme in this pathway, catalyze the phenylalanine to cinnamaic acid and then to trans-2-hydroxy-cinnamate making carbon flow enter into phenylpropanoid biosynthesis from shikimate pathway [86]. By the combination analysis of trascriptome studies, one *PAL* gene (AH19G32630) was identified significantly up-regulated which has been reported involving in biotic and abiotic stress [87–89]. Together with 4CL, they can catalyze the biosynthesis of p-coumaroyl-CoA. One *4CL* gene (AH13G11340) was identified obviously increased transcript abundance in P deficiency group suggesting that the encoded enzyme may be crucial for downstream metabolites responding to P deficiency stress. Lignin formation was one major downstream branch of the phenylpropanoid pathway. The three basic monolignols of lignin polymers included p-hydroxyphenyl (H), guaiacyl (G) and syringyl (S), which were derivated from p-coumaryl alcohols, coniferyl alcohols and sinapyl alcohols, respectively. The accumulation of sinapyl alcohol was increased in low P stress. CCR catalyzed the reaction to produce p-coumaraldehyde, caffeyl aldehyde, coniferyl dehyde, 5-hydeoxy-coniferaldehyde, and sinapaldehyde. One *CCR* gene (AH13G27210) has been identified significantly up- regulated under low P stress. CAD leaded the biosynthesis of p-coumaryl alcohol, caffeyl alcohol, coniferyl alcohol, 5-hydroxy-coniferyl alcohol, and sinapyl alcohol. The expression level of one *CAD* gene (AH20G03760) was remarkably increase in low P group. POD catalyzed the final step for the formation of lignin. The activity of POD also raised under P deficiency stress. Caffeic acid could be catalyzed by 4CL, CCR, CAD, and POD to involve the synthesis of guaiacyl lignin, 5-hydroxy-guaiacyl lignin, and syringyl lignin. Consequently, the increased accumulation of both caffeic acid and sinapyl alcohol provided a hint of raised lignin during low P stress. Lignin was the second organic carbon polymer on the earth. According to previous studies, lignin was indispensable for mechanical support of both microscopic cell wall and macroscopic plant xylem vessels which was vital for nutrients long distance transportation [90]. The increased metabolic flux of lignin synthesis pathway benefitted the plant development and adaption [91, 92]. Further, lignin has contributions to abiotic stresses response in plant [93–95]. The increased transcript level of *PAL, CAD, 4CL* genes could cause the accumulation of lignin to enhance tolerance [95–97]. The results of this study were consistent with previous reports. The up-regulated transcripts of lignin synthesis genes for instance of *PAL, 4CL, CCR, CAD* and *POD* genes may result in the accumulation of lignin and consequently increase plant acclimation to P deficiency condition. Under P nutrient limiting stress, accumulation of lignin could thickening and reduce permeability of cell wall, which helps plant to raise the adaptability against P deficiency stress. Moreover, phenylpropanoid pathway could be modulated by not only transcription regulation, but also plant hormone signal [98]. JA, a lipid derived hormone biosynthesized from α-linolenic acid metabolism responding to abiotic stress in plant, positively regulated the phenylpropanoid biosynthesis and increased the expression level of *PAL, 4CL* [99]. Further, it has been reported that endogenous both JA and 12-OPDA accumulation would promote root adaptation to stress conditions [43, 44, 100, 101]. As the results show, under low P condition, accumulation of 12-OPDA increased the production of JA in roots. The results indicated that JA signal may also take part in the regulation of P deficiency adaption.

In addition, under P deficiency condition, many kinds of amino acids were increased remarkably including two aromatic amino (phenylalanine, tryptophan), two BCAAs (leucine, isolcucine), three alkaline amino acids (lysine, histidine, arginine), threonine, and aspartate. The results provided a clue that the higher abundance of amino acids through C flow into N metabolism may play a critical function in P deficiency tolerance. Both under salt, Pb toxicity, and drought, the osmotic protection of amino acids have been reported [102–104]. In our study, increased content of amino acids may improving stress resistance by reduce permeability. Furthermore, as the crucial connector of shikimate pathway and phenylpropanoid biosynthesis, the accumulation of phenylalanine and tryptophan point to the biosynthesis of antioxidants [105]. Levan, a fructose polymer, was biosynthesized from sucrose metabolism. The higher accumulation of intracellular levan could improve the resistance of plant by protecting plasma membrane [106] and enhance the antioxidant capacity [107]. However, level of LPC down-regulated under P deficiency stress. Apparently, P nutrient was the important constituent elements of LPC. P deficiency may be the main reason for the reduction of LPC. In addition, another vital function of LPC was to activate specifically a proton pump H^+^-ATPase in plasma membrane, which was an important phosphate transporter on cell membrane [108]. Therefore, the decreased content of LPC may changed the dynamic structure and physical state of membrane causing decreased permeability of plasma [109]. From the above results, we can draw a conclusion that P deficiency has a great impact on cell membrane and cytoplasm. By increasing the content of lignin, amino acids, and levan combining with decreasing the content of LPC, cells maintained proper permeability for survival.

## Conclusions

**Fig. 8:**
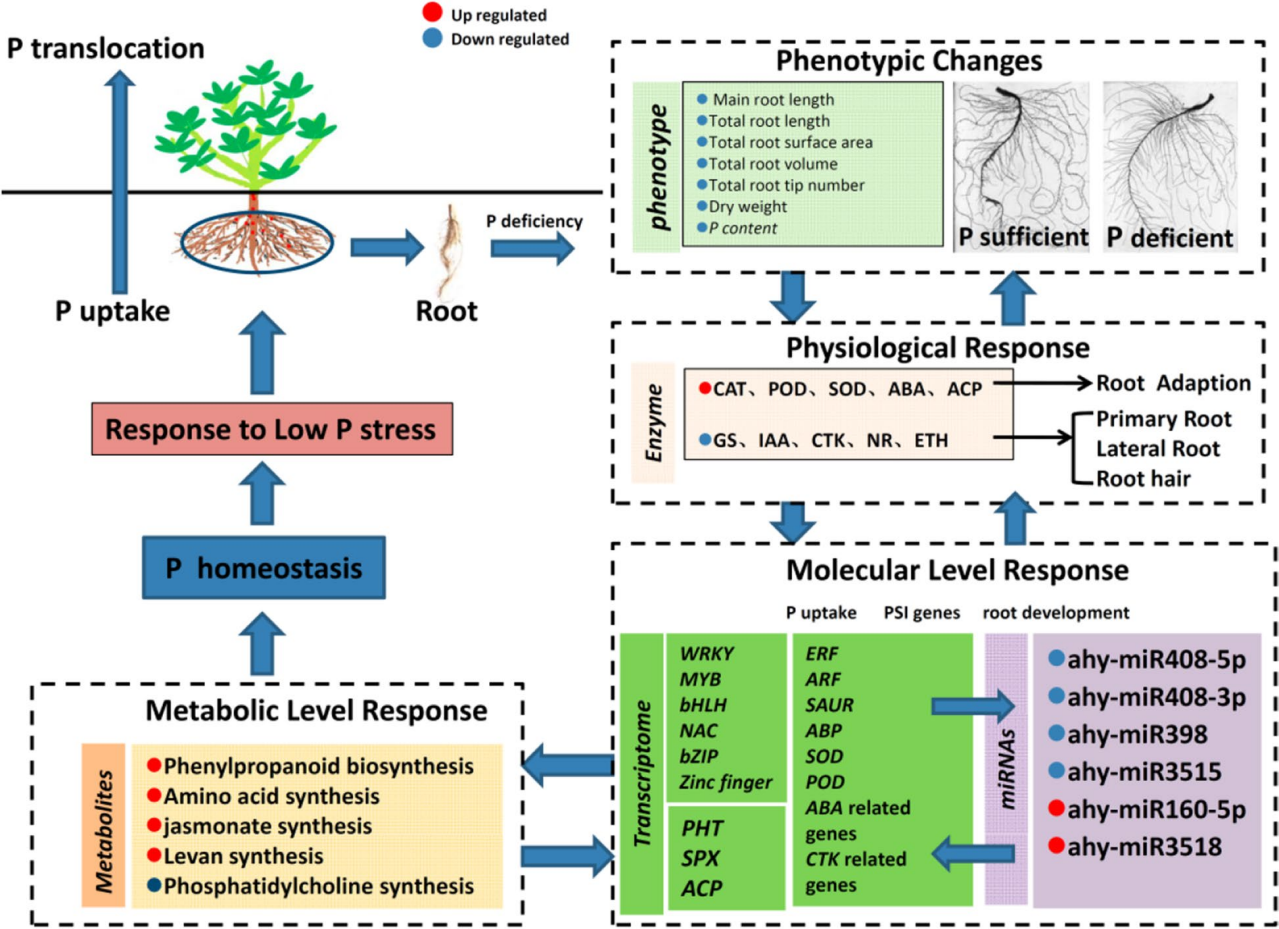
A summary of responding and regulatory mechanism involving DEGs, differentially expressed miRNAs, and DAMs in peanut root during P deficency

**Fig. 9:**
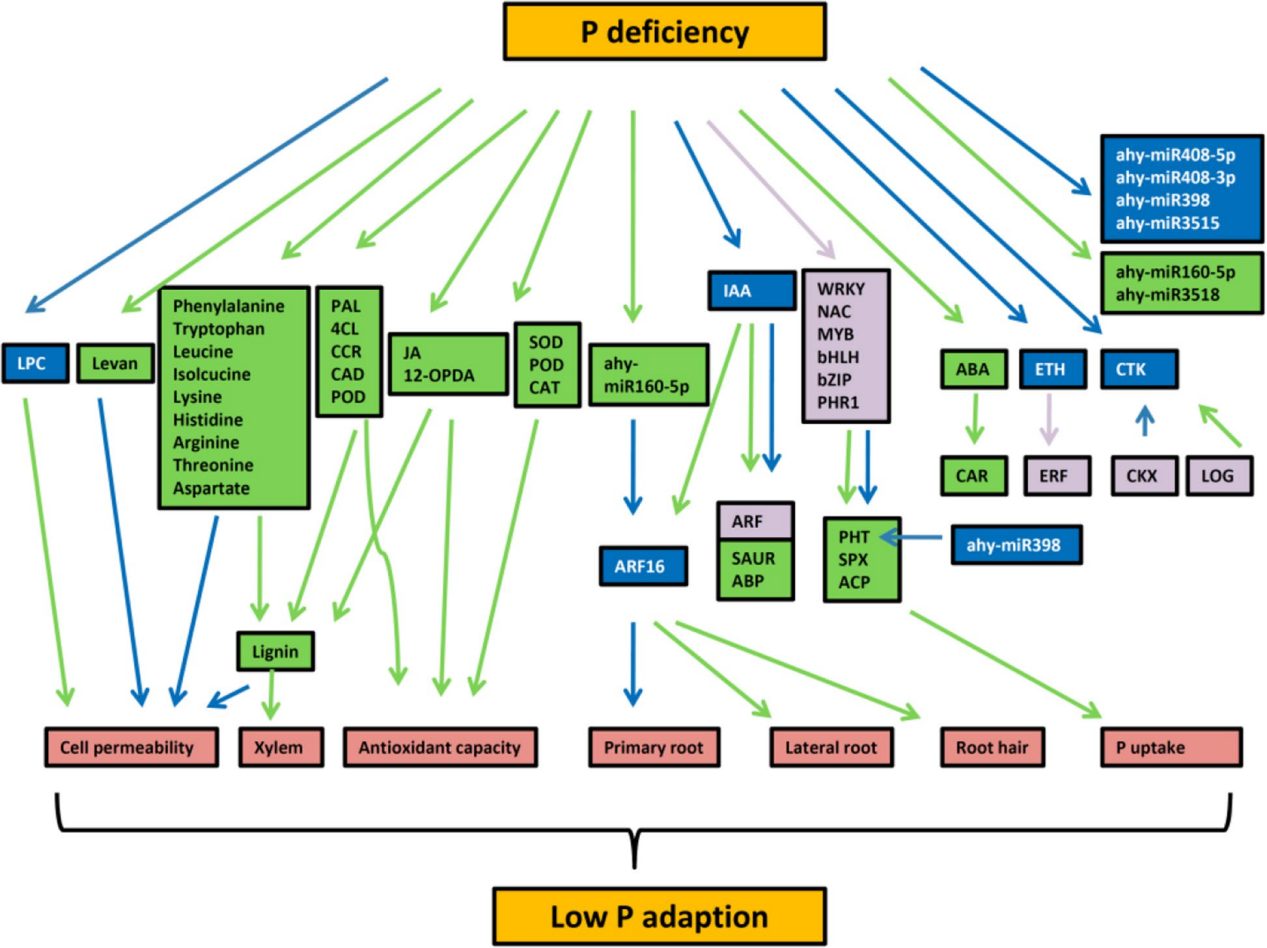
A proposed transcriptional and posttranscriptional regulation of peanut roots under P deficiency stress. Green arrow means positive regulation; blue arrow means negative regulation; purple means both. Green box means increasing; blue box means decreasing; purple means both

The tolerance of P deficiency of peanut root was an orchestrated regulated process through multiple metabolic biosynthesis pathways and transcriptional regulation. To better understand the response of peanut root to P deficiency stress, we performed a integrative analysis of phenotype, transcriptome and metabolites changes in peanut roots under P deficiency stress. A summary of detailed main phenomenon and predicted regulation relationship among metabolisms and transcripts were given in Figs. 8 and 9. Although several crucial genes of DAMs biosynthesis have been identified in transcriptome, still many other genes in primary identified metabolic pathway were not significantly changed in transcriptome between groups. Metabolites were the products of various biological process playing a vital role in plant growth and development and the contents were finely regulated by exogenous and endogenous factors. The results in this study indicated that post-transcriptional level may play a more sophisticated role during P deficiency. In addition, the comprehensive analysis of multiomics not only drawn several conclusions consistent with previous studies but also uncovered and focused the remarkable importance of lignin synthesis pathway, reduction of cell permeability, maintaining stability of cell, raising the antioxidant capacity and increasing the P uptake in struggling for survival under P deficiency stress. These findings provided a systematical understanding for peanut P deficiency tolerance and will benefit for further in-depth research to elucidate the function of candidate genes and metabolites in P deficiency tolerance.

## Methods

### Plant materials and treatments

The cultivated peanut variety of Huayu 22 was a virginia type cultivar with high yield and high P use efficiency, which is one of the dominant varieties in the study area. All the seeds were naturally air dried before use. Plump seeds of same size were selected and rinsed 3 times in distilled water. Then, the seeds were pre-germinated in a beaker at 28 °C for 12 h. After 3 days of germination, 30 peanut seedlings of uniform size were randomly divided into two groups and transferred to two hydroponic treatments containing Hoagland nutrient solutions: (1) P in deficiency with no P application (–P treatment) and (2) P in sufficiency with 0.6 mM P application (+ P treatment) in the form of KH_2_PO_4_. The Hoagland nutrient solution of + P treatment included the macroelements (0.50 mM NH_4_NO_3_, 0.60 mM K_2_SO_4_, 0.65 mM MgSO_4_·7H_2_O, 0.10 mM KCl, 2.00 mM Ca(NO_3_)_2_·4H_2_O) and microelements (1 µM H_3_BO_3_, 1 µM MnSO_4_·H_2_O, 0.1 µM CuSO_4_·5H_2_O, 1 µM ZnSO_4_·7H_2_O, 0.005 µM (NH_4_)_6_Mo_7_O_24_·4H_2_O, 0.1 mM Fe-ethylenediaminetetraacetgic acid (EDTA)). The same amounts of N was input under –P treatment. The level of K was balanced by adjusting the amount of K_2_SO_4_ at –P treatment. The nutrient solutions were prepared with distilled water. The solutions were aerated using an oxygenation pump three times a day and replaced twice a week. The pre-germinated seedlings under two treatments were carried out in controlled climate room, which was maintained at 28 °C, with 16 h photophase (16,000 lx) and 8 h night cycle and relative humidity of 50%. The measurement was performed for 60 days after the treatment.

### Plant phenotype detection

The phenotypes of the two treatments (–P and + P) were evaluated, including the following characteristics after culturing in Hoagland nutrient solution for 60 days: main root length (cm), main stem height (cm), lateral branch length (cm), number of branches, number of main stem leaves, root dry weight (g), stem dry weight (g) and leaf dry weight (g). Three parts of root, stem and leaf were separated from each plant and packed in three individual paper bags which were put into the oven for steaming at 100 °C for 30 min and dried at 70 °C to a constant weight. DW of each part was recorded separately.

### Root system scan

The phenotypes of each plant root in two P-level Hoagland nutrient solutions were investigated on the 60^th^ day after planting. WinRHIZO root analysis software (Regent instruments Inc., Quebec, Canada) was used to detect the change in root phenotype under P deficiency stress, including the total root length (cm), the total root surface area (cm^2^), the total root volume (cm^3^) and the total root tip number.

### Leaf photosynthesis test

The CIRAS-3 portable photosynthesis system (PP SYSTEMS, MA, USA) was used to test the photosynthesis of each plant in the two treatments, including A (μmol CO_2_ m^–2^ s^–1^), Ci (μmol CO_2_ mol^–1^), E (mmol H_2_O m^–2^ s^–1^), and Gs (mmol H_2_O m^–2^ s^–1^). Leaf photosynthesis was tested on the third youngest fully expanded leaf on each plant. All detection was conducted in accordance with the standard instruction of the meter. The detection location was in the controlled climate room, and the time was measured between 9:00 to 11:00 in the morning.

### P content detection in peanut

Three samples were randomly taken from the two treatments. The molybdenum blue colorimetric method was used to detect the P content of different tissues of the sample. Samples were digested with sulfuric acid and hydrogen peroxide to convert various forms of P into orthophosphate. The orthophosphate could react with the molybdenum antimony antichromogenic agent and produce blue phosphomolybdenum. The absorption value of the blue solution was directly proportional to the P content and can be read by an ELISA reader.

### Observation of peanut roots

Samples of main root apex and basal of main root under two treatments were observed. For the resin sections, fresh samples were washed and fixed by using FAA solution in a penicillin bottle. The tissues were dehydrated with gradient alcohol (50%, 70%, 85%, 90%, 100%), replaced with xylene, and then immersed in paraffin wax (60ºC). The tissue was embedded in paraffin wax block in HistoCore Arcadia H (Leica Biosystem, Wetzlar, Germany). Sections were cut to 4 μm thickness by a HistoCore biocut slicer (Leica Biosystem) and placed into xylene and gradient alcohol for dewaxing. Then, sections were stained with safranin (0.1%) dye solution with 3 min for each one. Sections were immersed in 95% alcohol for 15 s, then dehydrated with absolute ethanol, the transparent xylene, then air-dried, and sealed with neutral gum to make permanent sections. Sections were observed, and then photos were taken under an Olympus IX73P + DP80 microscope at a magnification of 200 times (Olympus, Tokyo, Japan).

### Enzyme activity determination

The soluble enzyme solution was extracted from peanut roots. Fresh roots (0.5 g) were ground by pestle in a mortar with 6 mL of 0.2 M ice precooled potassium phosphate (KH_2_PO_4_ and K_2_HPO_4_) buffer (pH 7.8). The homogenate was centrifuged at 4000 g for 20 min. The supernatant (enzyme solution) was put into the test tube for the determination of enzyme activities. All procedures were carried out at 4ºC. The 10 enzyme activities were tested from the enzyme solution above by using an ELISA kit (MDBio, Taipei, China) following the standard protocols for each enzyme, including SOD, GS, POD, IAA, ACP, ABA, CTK, NR, ETH, and CAT. The activities were evaluated as IU mL^−1^ on an iMark Microplate Absorbance Reader (BioRad, Hercules, CA, USA).

### Transcriptome sequencing to identify the DEGs in low P stress

Total RNA was extracted from roots tissues of Huayu 22 peanut that was hydroponically grown in total nutrient (0.6 mM Pi, + P treatment) and non P nutrient (0 mM Pi, –P treatment) Hoagland nutrient solution for 60 days. The RNA-Seq transcriptome library was constructed, and the sequencing was commissioned. The transcriptome sequencing platform was an Illumina Novaseq 6000 in Majorbio company (Shanghai, China), with 3 biological replicates for each treatment and 3 experimental repetitions for each sample. According to the expression level of each sample, the DESeq2 method was used to detect DEGs between –P and + P treatments. The qRT-PCR was conducted to validate the results of gene expression level. There were 10 DEGs selected for qRT-PCR validation. Specific primers were designed using Primer Premier v5.0. Total RNAs from roots were extracted by Trizol reagent according to the instructions (Invitrogen, CA, USA). cDNA was synthesized with oligo (dT) primer and RNase-free DNase treated RNA sample with SuperScript First-Strand synthensis system for qRT-PCR (Invitrogen, USA). The PCR was performed using 2 × SYBR Green Master Mix (Takara, Japan) with three biological replicates and three technical replicates. The qRT-PCR assay was carried out in a 7500 Fast Real Time PCR system (Appliedbiosystems, USA). *AhTUA5* gene was used as the endogenous reference to normalize the expression level of target genes. The relative expression level of DEGs were analyzed by 2 ^−△△CT^ methods [110]. The primers used in the study were listed in Supplemental Table S5.

### High-throughput sequencing of miRNAs in response to low P stress

Root tissues of three treated plants (–P) and three control plants (+ P) were used to identify miRNAs involving in peanut low P stress. Total RNA was extracted by TRIZOL reagent (Invitrogen, Carlsbad, CA, USA) with the elimination of genomic DNA by DNase I (Tiangen Biotech, Beijing, China). Peanut RNA-seq miRNA libraries were constructed by Illumina TruSeq Small RNA kit (Invitrogen) and the sequencing by Illumina Hiseq 4000 platform with 3 biological replicates for each treatment and 3 experimental replicates. Total 67.62 M clean reads were assembled according to the reference genome sequence of peanut cultivar Shitouqi (PGR: http://peanutgr.fafu.edu.cn/Download.php) by Bowtie (http://bowtie-bio.sourceforge.net/index.shtml). The screening criteria was p-adjust < 0.05 and |log_2_FC|≥ 1. TPM was used to calculate the expression level. The DESeq2 method was used to compare the differentially expressed genes (DEGs) between –P and + P group. The target genes of miRNAs were predicted with psRobot (http://omicslab.genetics.ac.cn/psRobot/index.php). GO and KEGG analysis were conducted based on GO and KEGG database. To further understand the regulatory network of miRNAs, precursor miRNA (pre-miRNA) sequences were conducted for BLAT with peanut genome sequences and 2000 bp upstream regions were retrieved. Pre-miRNAs were transcribed by RNA polymerase II which can recognize class II promoters. Therefore, 2000 bp upstream of each miRNA gene was scanned for TSSs and *cis*-elements by TSSPlant (http://softberry.com) and NSITE-PL software [111], respectively. Cytoscape 3.9.1 was used to analysis the network of miRNA and target genes [112]. To verify the results of high-throughput sequencing, 5 microRNAs and 5 target genes were selected for qRT-PCR. Reverse transcription was conducted by miRNA First Strand cDNA Synthesis Tailing Reaction Kit (Sangon Biotech, Shanghai, China) following the instructions. The reaction of qRT-PCR was performed using miRNA qRT-PCR SYBR Kit (Clontech, CA). *U6* was used as the reference gene. The relative expression level of target genes were detected using the same method described in transcriptome validation and normalized to the endogenous control *AhTUA5*. All primers were showed in Supplemental Table S5.

### Analysis for identification of differentially accumulated metabolites in low P stress

Samples used for metabolite analysis were the same as transcriptome analysis. The metabolite analysis were also conducted with peanut roots collecting from P deficient and P sufficient group, respectively. About 100 mg frozen roots tissue were grounded into powder in liquid nitrogen. 50 mg fine weighed powder were suspended with 500 µL extraction liquid (methanol: water = 4:1, v/v). After vortex, the mixtures were settled on ice and then treated with ultrasound at 40 kHz for 30 min at 5 °C. Following centrifuged at 15,000 rpm at 4 °C for 15 min, the supernatant were carefully transferred to a fresh EP tube and injected into LC–MS/MS instrument platform (UHPLC-Q Exactive system of Thermo Fisher Scientific) for analysis. After injection 2 µL samples onto HSS T3 column (100 mm × 2.1 mm), samples were detected by mass spectrometry detection. The solvent A mobile phase consisted of 0.1% formic acid in water: acetonitrile (95: 5, v/v) and solvent B included 0.1% formic acid in acetonitrile: isopropanol: water (47.5: 47.5: 5, v/v/v) with solvent gradient at the flow rate of 0.4 mL min^−1^ as follows: from 0 to 0.1 min, 0% B—5% B; from 0.1 to 2 min, 5% B—25% B; from 2 to 9 min, 25% B—100% B; from 9 to 13 min, 100% B; from 13 to 13.1 min, 100% B—0% B; from 13.1 to 16 min, 0% B for equilibrating the systems. A Thermo UHPLC-Q Exactive Mass Spectrometer equipped with an electrospray ionization (ESI) source were set as follows: heater temperature of 400 °C; capillary temperature of 320 °C; sheath gas flow rate of 40 arb; aux gas flow rate of 10 arb; ion-spray voltage floating (ISVF) of 3500 V in positive mode and -2800 V in negative mode, respectively. The raw data was preprocessed by Progenesis QI software (Waters Corporation, Milford, USA). Metabolites were identified according to the HMDB (http://www.hmdb.ca/), Metlin (https://metlin.scripps.edu/) and Majorbio Database.

### Statistical analysis

A completely randomized design (CRD) with three repetitions was used for each test. Data were analyzed by statistical software SPSS 22.0. The differences between the two treatments were compared using the Duncan method (new multiple range method) at the significance level of *p* < 0.05 or* p* < 0.01.

## Supplementary Information

**Additional file 1: Supplemental Dataset S1.** All differentially expressed genes between two P levels.

**Additional file 2: Supplemental Dataset S2.** A total of 439 DAMs between two P levels.

**Additional file 3: Supplemental Fig. S1.** Effect of phosphorus on activities of related enzymes and hormone in peanut leaves. * indicates *P* < 0.05. ** indicates *P* < 0.01. **Supplemental Fig. S2.** Differentially expressed gene (DEGs) clustering. (A) Heat map of differentially expressed gene. Transformed TPM (Transcripts Per Million reads) values were used to measure expression level. Red color represents the high expression level; and blue color represents the low expression level. C1-C3 indicates +P group; T1-T3 indicates –P group. (B) The red dots indicates the significantly up-regulated genes, and the green dots indicates the significantly down-regulated genes. **Supplemental Fig. S3.** Verification and consistency analysis of the selected genes expression level between RNA-Seq and qRT-PCR results in peanut roots. **Supplemental Fig. S4.** Sequence lengths distribution of obtained miRNAs by high throughput sequencing in each sample. C1-C3 indicates +P group; T1-T3 indicates –P group. **Supplemental Fig. S5.** Top15 expressed miRNAs (A) and expression distribution (B) in each sample. **Supplemental Fig. S6.** qRT-PCR verification and consistency analysis of the selected miRNAs and target genes in peanut roots. **Supplemental Fig. S7.** OPLS-DA analysis (A) and heat map of DAMs clustering. C1-C3 indicates +P group; T1-T3 indicates –P group. **Supplemental Fig. S8.** Differentially accumulated metabolites (DAMs) between treatments. (A) Volcano plot showing the DAMs between two P levels. The red dots indicates the significantly increased DAMs, the green dots indicates the significantly decreased DAMs. (B) PCA analysis of samples. (C) Correlation analysis between samples. (D) Correlation analysis of DAMs. **Supplemental Fig. S9.** Compounds classification and function analysis. (A) Compounds classification by KEGG Compound database. (B) Compounds classification by HMDB database. (C) KEGG pathway classification. (D) KEGG enrichment analysis of DAMs.

**Additional file 4: Supplemental Table S1.** Phenotypic differences of roots between two P level treatments.

**Additional file 5: Supplemental Table S2.** Original data of transcriptome sequencing.

**Additional file 6: Supplemental Table S3.** Information of DEGs related to P deficiency stress of transcriptome analysis.

**Additional file 7: Supplemental Table S4.** Original data of miRNA sequencing.

**Additional file 8: Supplemental Table S5.** List of primer sequences used in qRT-PCR verification.

## Data Availability

Transcriptome sequencing data are available in the SRA database of National Center for Biotechnology Information (NCBI) under the accession number of BioSample SAMN27190916-SAMN27190921, and BioProject PRJNA822521 (https://www.ncbi.nlm.nih.gov/sra/PRJNA822521). The NCBI SRA accession numbers of miRNA data files were BioSample SAMN28859603-SAMN28859608, and BioProject PRJNA845220 (https://www.ncbi.nlm.nih.gov/sra/PRJNA845220). The raw data files of metabolome were deposited to China National GeneBank DataBase (CNGBdb) with accession number CNP0003314 (http://db.cngb.org/cnsa/project/CNP0003314_ff031411/reviewlink/).

## Abbreviations

4CL: 4-Coumarate-CoA ligase
A: Net photosynthetic rate
ABA: Abscisic acid
ABP: Auxin binding protein genes
ACP: Acid phosphatase
ARF: Auxin response factor
AuxRE: Auxin response element
CAD: Cinnamyl alcohol dehydrogenase
CAR: C2 domain ABA related gene
CAT: Catalase
CCR: Cinnamoyl-CoA reductase
Ci: Intercellular CO_2_ concentration
CKX: Cytokinin dehydrogenase
CO: Cortex
CTK: Cytokinin
DAM: Differentially accumulated metabolite
DEG: Differentially expressed gene
DW: Dry weight
E: Transpiration rate
ELISA: Enzyme-linked immunosorbent assay
EP: Epidermis
ERF: Ethylene response transcription factors
ETH: Ethylene
FAA: Formalin-acetic acid–ethanol
FDR: False discovery rate
GlpT1: Glycerol-3-phosphate transporter 1
GO: Gene Ontology
GS: Glutamine synthetase
Gs: Stomatal conductance
IAA: Indole-3-acetic acid
JA: Jasmonic acid
K: Potassium
KEGG: Kyoto Encyclopedia of Genes and Genomes
LOG: Cytokinin riboside 5'-monophosphate phosphoribohydrolase
N: Nitrogen
NR: Nitrate reductase
P: Phosphorus
P1BS: PHR1-binding sequence
PAL: Phenylalanine ammonia lyase
PCA: Principal component analysis
PFP1: Pyrophosphate-fructose 6-phosphate 1-phosphotransferase
PHR1: Phosphate starvation response 1
PHT: Phosphate transporter
POD: Peroxidase
PP: Parenchyma pith
PSR: P starvation responsive
PYL: Pyrabactin resistance-like protein
qRT-PCR: Quantitative reverse-transcription PCR
ROS: Reactive oxygen species
SAUR: Small auxin upregulated RNA
SOD: Superoxide dismutase
SPH: Secondary phloem
SXY: Secondary xylem
TCA cycle: Citrate cycle
TF: Transcription factor
TFBS: Transcription factor binding site
TPM: Transcripts per million reads
TSS: Transcription start site

## References

[1] Taru VB, Kyagya IZ, Mshelia SI, Adebayo EF (2008). Economic efficiency of resource use in groundnut production in Adamawa state of Nigeria post primary schools management board yola, Adamawa state. Nigeria World J Agric Sci.

[2] Veeramani P, Subrahmaniyan K (2011). Nutrient management for sustainable groundnut productivity in India-a review. Int J Adv Sci Eng Inf Technol.

[3] Pourranjbari SS, Souri MK, Moghaddam M (2019). Characterization of nutrients uptake and enzymes activity in Khatouni melon (Cucumis melo var. inodorus) seedlings under different concentrations of nitrogen, potassium and phosphorus of nutrient solution. J Plant Nutr..

[4] Yu TY, Wang CX, Sun XW, Wu ZF, Zheng YM, Sun XS, Shen P, Wang CB (2016). Characteristics of phosphorus and dry matter accumulation and distribution in peanut cultivars with different yield and phosphorus use efficiency. Chin J Oil Crop Sci.

[5] Wang YF, Xu L, Zhao CX, Wang ML (2012). Effects of phosphorus application on nitrogen accumulation sources and yield of peanut. Chin J Soil Sci.

[6] Fairhust T, Lefroy R, Mutert E (1999). The importance, distribution and cause of phosphorus deficiency as a constraint to crop production in the tropics. Agroforestry-forestry Forum.

[7] Cui BW, Qiao G, Fan FH, Ding GJ, Wen XP (2017). Principal component analysis and grey correlation analysis for low phosphorus tolerence evaluation in masson Pine (Pinus massoniana) of different provenances. J Southwest Univ (Natural Science Edition).

[8] Guan JY, Fan WG (2018). Effect of phosphorus supply on growth, nutrient content and related physiology indexes of Rosa roxburghii seedlings. Acta Bot Boreali-Occidentalia Sin.

[9] Brown LK, George TS, Thompson JA, Wright G, Lyon J, Dupuy L, Hubbard SF, White PJ (2012). What are the implications of variation in root hair length on tolerance to phosphorus deficiency in combination with water stress in barley (Hordeum vulgare). Ann Bot.

[10] Bustos R, Castrillo G, Linhares F, Puga MI, Rubio V, Pérez-Pérez J, Solano R, Leyva A, Paz-Ares J (2010). A central regulatory system largely controls transcriptional activation and repression responses to phosphate starvation in Arabidopsis. Plos Genet.

[11] Deng Y, Men C, Qiao S, Wang W, Gu J, Liu L, Zhang Z, Zhang H, Wang Z, Yang J (2020). Tolerance to low phosphorus in rice varieties is conferred by regulation of root growth. Crop J.

[12] Chen SS, Ding GD, Wang ZH, Cai HM, Xu FS (2015). Proteomic and comparative genomic analysis reveals adaptability of Brassica napus to phosphorus-deficient stress. J Proteomics.

[13] Vasconcelos MJV, Figueiredo JEF, Raghothama KG (2018). Expression analysis of anthocyanin gene induced under phosphorus starvation in maize genotypes with contrasting phosphorus use efficiency. Genet Mol Res.

[14] Wei ZQ, Shi YX, Kong FM (2002). The effect of phosphorus deficiency stress on acid phosphatase in peanut. Chin J Oil Crop Sci.

[15] Veronica N, Subrahmanyam D, Kiran TV, Yugandhar P, Bhadana VP, Padma V, Jayasree G, Voleti SR (2017). Influence of low phosphorus concentration on leaf photosynthetic characteristics and antioxidant response of rice genotypes. Photosynthetica.

[16] Zhang K, Liu H, Tao P, Chen H (2014). Comparative proteomic analyses provide new insights into low phosphorus stress responses in maize leaves. PLoS ONE.

[17] Snezhkina AV, Kudryavtseva AV, Kardymon OL, Savvateeva MV, Melnikova NV, Krasnov GS, Dmitriev AA (2019). ROS Generation and antioxidant defense systems in normal and malignant cells. Oxid Med Cell Longev.

[18] Hammond JP, White PJ (2008). Sucrose transport in the phloem: integrating root responses to phosphorus starvation. J Exp Bot.

[19] Shen P, Luo S, Wu ZF, Sun XS, Wang CB, Yu TY, Zheng YM, Sun XW, Zheng YP (2015). Response of P absorption-allocation rate and root morphology of peanut to P foliar fertilizers with different acidities. J Nucl Agric Sci.

[20] Shi Q, Pang J, Wan J, Yong H, Bai C, Pereira CG, Song Q, Wu D, Dong Q, Cheng X, Wang F, Zheng J, Liu Y, Lambers H (2020). Phosphorus-fertilisation has differential effects on leaf growth and photosynthetic capacity of Arachis hypogaea L. Plant soil.

[21] Hernández I, Munné-Bosch S (2015). Linking phosphorus availability with photo-oxidative stress in plants. J Exp Bot.

[22] Li M, Yu B (2021). Recent advances in the regulation of plant miRNA biogenesis. RNA Biol.

[23] Millar AA (2020). The Function of miRNAs in plants. Plants (Basel).

[24] Meyers BC, Axtell MJ (2019). MicroRNAs in Plants: Key findings from the early years. Plant Cell.

[25] Basso MF, Ferreira P, Kobayashi AK, Harmon FG, Nepomuceno AL, Molinari HBC, Grossi-de-Sa MF (2019). MicroRNAs and new biotechnological tools for its modulation and improving stress tolerance in plants. Plant biotechnol.

[26] Pagano L, Riccardo R, Paesano L, Marmiroli N, Marmiroli M (2021). miRNA regulation and stress adaptation in plants. Environ Experiment Bot.

[27] Xin W, Zhang LN, Zhang WZ, Gao JP, Yi J, Zhen XX, Du M, Zhao YZ, Chen LQ (2019). An integrated analysis of the rice transcriptome and metabolome reveals root growth regulation mechanisms in response to nitrogen availability. Int J Mol Sci.

[28] Mao J, Huang L, Chen M, Zeng W, Feng Z, Huang S, Liu T (2021). Integrated Analysis of the transcriptome and metabolome reveals genes involved in terpenoid and flavonoid biosynthesis in the Loblolly Pine (Pinus taeda L.). Front Plant Sci..

[29] Guo M, Ruan W, Li C, Huang F, Zeng M, Liu Y, Yu Y, Ding X, Wu Y, Wu Z, Mao C, Yi K, Wu P, Mo X (2015). Integrative comparison of the role of the phosphate response1 subfamily in phosphate signaling and homeostasis in rice. Plant Physiol.

[30] Xu L, Zhao H, Wen R, Liu Y, Xu Z, Tian W, Ruan W, Wang F, Deng M, Wang J, Dolan L, Luam S, Xue S, Yi K (2019). Identification of vacuolar phosphate efflux transporters in land plants. Nat Plants.

[31] Sun W, Ma Z, Chen H, Liu M (2020). Genome-wide investigation of WRKY transcription factors in Tartary buckwheat (Fagopyrum tataricum) and their potential roles in regulating growth and development. PeerJ.

[32] Liu TY, Huang TK, Yang SY, Hong YT, Huang SM, Wang FN, Chiang SF, Tsai SY, Lu WC, Chiou TJ (2016). Identification of plant vacuolar transporters mediating phosphate storage. Nat Commun.

[33] Liu J, Yang L, Luan M, Wang Y, Zhang C, Zhang B, Shi J, Zhao FG, Lan W, Luan S (2015). A vacuolar phosphate transporter essential for phosphate homeostasis in Arabidopsis. Proc Natl Acad Sci USA.

[34] Secco D, Wang C, Arpat BA, Wang Z, Poirier Y, Tyerman SD, Wu P, Shou H, Whelan J (2012). The emerging importance of the SPX domain-containing proteins in phosphate homeostasis. New Phytol.

[35] Licausi F, Ohme-Takagi M, Perata P (2013). APETALA2/Ethylene responsive factor (AP2/ERF) transcription factors: mediators of stress responses and developmental programs. New Phytol.

[36] Rodriguez L, Miguel GG, Maira D, Americo R, Ana CIG, Marta PL, Maria AF, Regina A, Daniel F, Jose AM, Jose MM, Armando A, Pedro LR (2014). C2-domain abscisic acid-related proteins mediate the interaction of PYR/PYL/RCAR abscisic acid receptors with the plasma membrane and regulate abscisic acid sensitivity in Arabidopsis. Plant Cell.

[37] Xie FL, Huang SQ, Guo K, Zhu YY, Nie L, Yang ZM (2007). Computational identification of novel microRNAs and targets in Brassica napus. FEBS Lett.

[38] Gao GY, Wu XF, Huang W, Zhou DG, Zhang DW, Zhou ML, Zhang KX, Yan ML (2020). Regulation of flavonoid pathway by BjuB.KAN4 gene in Brassica juncea. Acta Agronomica Sinica..

[39] Yoshida S, Ito M, Nishida I, Watanabe A (2001). Isolation and RNA gel blot analysis of genes that could serve as potential molecular markers for leaf senescence in Arabidopsis thaliana. Plant Cell Physiol.

[40] Kizis D, Lumbreras V, Pagès M (2001). Role of AP2/EREBP transcription factors in gene regulation during abiotic stress. FEBS lett.

[41] Zhu YF, Huang PC, Guo PC, Chong L, Yu GB, Sun XL, Hu T, Li Y, Hsu CC, Tang K, Zhou Y, Zhao CZ, Gao W, Tao A, Mengiste T, Zhu JK (2020). CDK8 is associated with RAP2.6 and SnRK2.6 and positively modulates abscisic acid signaling and drought response in Arabidopsis. New Phytol..

[42] Kim P, Xue CY, Song HD, Gao Y, Feng L, Li Y, Xuan YH (2021). Tissue-specific activation of DOF11 promotes rice resistance to sheath blight disease and increases grain weight via activation of SWEET14. Plant Biotechnol.

[43] Griffiths G (2020). Jasmonates: biosynthesis, perception and signal transduction. Essays Biochem.

[44] Han X, Zhang M, Yang M, Hu Y (2020). Arabidopsis JAZ Proteins interact with and suppress RHD6 transcription factor to regulate Jasmonate-stimulated root hair development. Plant Cell.

[45] Van den Ende W, Coopman M, Clerens S, Vergauwen R, Le Roy K, Lammens W, Van Laere A (2011). Unexpected presence of graminan-and levan-type fructans in the evergreen frost-hardy eudicot Pachysandra terminalis (Buxaceae). Purification, cloning and functional analysis of a 6-SST/6-SFT enzyme. J Plant Physiol..

[46] Peñuelas J, Poulter B, Sardans J, Ciais P, van der Velde M, Bopp L, Boucher O, Godderis Y, Hinsinger P, Llusia J, Nardin E, Vicca S, Obersteiner M, Janssens IA (2013). Human-induced nitrogen-phosphorus imbalances alter natural and managed ecosystems across the globe. Nat Commun..

[47] Rouached H, Arpat AB, Poirier Y (2010). Regulation of phosphate starvation responses in plants: signaling players and cross-talks. Mol Plant.

[48] Yang H, Chen R, Chen Y, Li H, Wei T, Xie W, Fan G. Agronomic and physiological traits associated with genetic improvement of phosphorus use efficiency of wheat grown in a purple lithomorphic soil. Crop J. 2022;10:1151–64.

[49] Zhang W, Li H, Zhang J, Shen J, Brown H, Wang E (2022). Contrasting patterns of accumulation, partitioning, and remobilization of biomass and phosphorus in a maize cultivar. Crop J.

[50] Zheng YP, Xin CY, Wang CB, Sun XS, Yang WQ, Wan SB, Zheng YM, Feng H, Chen DX, Sun XW, Wu ZF (2013). Effects of phosphorus fertilizer on root morphology, physiological characteristics and yield in peanut (Arachis hypogaea). Chin J Plant Ecol.

[51] Karthikeyan AS, Varadarajan DK, Jain A, Held MA, Carpita NC, Raghothama KG (2007). Phosphate starvation responses are mediated by sugar signaling in Arabidopsis. Planta.

[52] Ligaba A, Yamaguchi M, Shen H, Sasaki T, Yamamoto Y, Matsumoto H (2004). Phosphorus deficiency enhances plasma membrane H^+^-ATPase activity and citrate exudation in greater purple lupin (Lupinus pilosus). Funct Plant Biol.

[53] Shen H, Chen J, Wang Z, Yang CY, Sasaki T, Yamamoto Y, Matsumoto H, Yan XL (2006). Root plasma membrane H^+^-ATPase is involved in the adaptation of soybean to phosphorus starvation. J Exp Bot.

[54] Kavanová M, Grimoldi AA, Lattanzi FA, Schnyder H (2006). Phosphorus nutrition and mycorrhiza effects on grass leaf growth. P status- and size-mediated effects on growth zone kinematics. Plant Cell Environ..

[55] He G, Zhang J, Hu X, Wu J (2011). Effect of aluminium toxicity and phosphorus deficiency on the growth and photosynthesis of oil tea (Camellia oleifera Abel.) seedlings in acidic red soils. Acta Physiol Plant..

[56] Assuero SG, Mollier A, Pellerin S (2004). The decrease in growth of phosphorus-deficientmaize leaves is related to a lower cell production. Plant Cell Environ.

[57] Zhang Z, Liao H, Lucas WJ (2014). Molecular mechanisms underlying phosphate sensing, signaling, and adaptation in plants. J Integr Plant Biol.

[58] Rohman MM, Talukder MZA, Hossain MG, Uddin MS, Amiruzzaman M, Biswas A, Ahsan AFMS, Chowdhury MAZ (2016). Saline sensitivity leads to oxidative stress and increases the antioxidants in presence of proline and betaine in maize (Zea mays L.) inbred. Plant Omics..

[59] Hasanuzzaman M, Alam MM, Rahman A, Hasanuzzaman M, Nahar K, Fujita M (2014). Exogenous proline and glycine betaine mediated upregulation of antioxidant defense and glyoxalase systems provides better protection against salt-induced oxidative stress in two rice (Oryza sativa L.) varieties. Biomed Res Int..

[60] Castro-Rodríguez V, García-Gutiérrez A, Canales J, Avila C, Cánovas FM (2011). The glutamine synthetase gene family in Populus. BMC Plant Biol.

[61] Tanimoto M, Roberts K, Dolan L (1995). Ethylene is a positive regulator of root hair development in Arabidopsis thaliana. Plant J.

[62] Vissenberg K, Claeijs N, Balcerowicz D, Schoenaers S (2020). Hormonal regulation of root hair growth and responses to the environment in Arabidopsis. J Exp Bot.

[63] Zhang H, Zhu J, Gong Z, Zhu J (2022). Abiotic stress responses in plants. Nat Rev Genet.

[64] Neill SJ, Desikan R, Hancock JT (2003). Nitric oxide signaling in plants. New Phytol.

[65] Deng QW, Luo XD, Chen YL, Zhou Y, Zhang FT, Hu BL, Kun J (2018). Transcriptome analysis of phosphorus stress responsiveness in the seedlings of Dongxiang wild rice (Oryza rufipogon Griff.). Biol Res..

[66] Jiang M, Sun L, Isupov MN, Littlechild JA, Wu X, Wang Q, Wang Q, Yang W, Wu Y (2019). Structural basis for the target DNA recognition and binding by the MYB domain of phosphate starvation response 1. FEBS J.

[67] Zhang J, Gu M, Liang R, Shi X, Chen L, Hu X, Wang S, Dai X, Qu H, Li H, Xu G (2021). OsWRKY21 and OsWRKY108 function redundantly to promote phosphate accumulation through maintaining the constitutive expression of OsPHT1;1 under phosphate-replete conditions. New Phytol.

[68] Wang F, Ge S, Xu X, Xing Y, Du X, Zhang X, Lv M, Liu J, Zhu Z, Jiang Y (2021). Multiomics analysis reveals new insights into the apple fruit quality decline under high nitrogen conditions. J Agric Food Chem.

[69] Sharma A, Shahzad B, Rehman A, Bhardwaj R, Landi M, Zheng B (2019). Response of phenylpropanoid pathway and the role of polyphenols in plants under abiotic stress. Molecules.

[70] Lin WY, Huang TK, Leong SJ, Chiou TJ (2014). Long-distance call from phosphate: systemic regulation of phosphate starvation responses. J Exp Bot.

[71] Huang D, Koh C, Feurtado JA, Tsang EWT, Cutler AJ (2013). MicroRNAs and their putative targets in Brassica napus seed maturation. BMC Genomics.

[72] Liu J, Guo X, Zhai T, Shu A, Zhao L, Liu Z, Zhang S (2020). Genome-wide identification and characterization of microRNAs responding to ABA and GA in maize embryos during seed germination. Plant Biol.

[73] Wang JW, Wang LJ, Mao YB, Cai WJ, Xue HW, Chen XY (2005). Control of root cap formation by MicroRNA-targeted auxin response factors in Arabidopsis. Plant Cell.

[74] Mallory AC, Bartel DP, Bartel B (2005). MicroRNA-directed regulation of Arabidopsis auxin response factor17 is essential for proper development and modulates expression of early auxin response genes. Plant Cell.

[75] Sun SB, Gu M, Cao Y, Huang XP, Zhang X, Ai PH, Zhao JN, Fan XR, Xu GH (2012). A constitutive expressed phosphate transporter, OsPht1;1, modulates phosphate uptake and translocation in phosphate-replete rice. Plant Physiol.

[76] Kumar S, Verma S, Trivedi PK (2017). Involvement of Small RNAs in phosphorus and sulfur sensing, signaling and stress: Current Update. Front Plant Sci.

[77] Dugas DV, Bartel B (2008). Sucrose induction of Arabidopsis miR398 represses two Cu/Zn superoxide dismutases. Plant Mol Biol.

[78] Ye Y, Yuan J, Chang X, Yang M, Zhang L, Lu K, Lian XM (2015). The phosphate transporter gene OsPht1;4 is involved in phosphate homeostasis in rice. PLoS ONE.

[79] Liang G, Ai Q, Yu D (2015). Uncovering miRNAs involved in crosstalk between nutrient deficiencies in Arabidopsis. Sci Rep.

[80] Shahbaz M, Pilon M (2019). Conserved Cu-MicroRNAs in Arabidopsis thaliana function in copper economy under deficiency. Plants.

[81] Tobimatsu Y, Schuetz M (2019). Lignin polymerization: how do plants manage the chemistry so well?. Curr Opin Biotechnol.

[82] Dixon RA, Paiva NL (1995). Stress-induced phenylpropanoid metabolism. Plant Cell.

[83] Barros J, Shrestha H, Serrani-Yarce J, Engle N, Abraham P, Tschaplinski T, Hettich R, Dixon R (2022). Proteomic and metabolic disturbances in lignin-modified Brachypodium distachyon. Plant Cell.

[84] Fang Z, Shao C, Meng Y, Wu P, Chen M (2009). Phosphate signaling in Arabidopsis and Oryza sativa. Plant Sci.

[85] Sha A, Chen Y, Ba H, Shan ZH, Zhang XJ, Wu XJ, Qiu DZ, Chen SL, Zhou X (2012). Identification of Glycine Max MicroRNAs in response to phosphorus deficiency. J Plant Biol.

[86] Yu M, Man Y, Lei R, Lu X, Wang Y (2020). Metabolomics study of flavonoids and anthocyanin-related gene analysis in kiwifruit (Actinidia chinensis) and kiwiberry (Actinidia arguta). Plant Mol Biol Rep.

[87] Hamberger B, Ellis M, Friedmann M, de Azevedo SC, Barbazuk B, Douglas C (2007). Genome-wide analyses of phenylpropanoid-related genes in Populus trichocarpa, Arabidopsis thaliana, and Oryza sativa: the Populus lignin toolbox and conservation and diversification of angiosperm gene families. Can J Bot.

[88] Lillo C, Lea US, Ruoff P (2008). Nutrient depletion as a key factor for manipulating gene expression and product formation in different branches of the flavonoid pathway. Plant Cell Environ.

[89] Bhuiyan NH, Selvaraj G, Wei Y, King J (2009). Gene expression profiling and silencing reveal that monolignol biosynthesis plays a critical role in penetration defence in wheat against powdery mildew invasion. J Exp Bot.

[90] Zhao S, Zhao L, Liu F, Wu Y, Zhu Z, Sun C, Tan L (2016). Narrow and rolled leaf 2 regulates leaf shape, male fertility, and seed size in rice. J Integr Plant Biol.

[91] Wang JP, Matthews ML, Naik PP, Williams CM, Ducoste JJ, Sederoff RR, Chiang VL (2019). Flux modeling for monolignol biosynthesis. Curr Opin Biotechnol.

[92] Dong NQ, Lin HX (2021). Contribution of phenylpropanoid metabolism to plant development and plant-environment interactions. J Integr Plant Biol.

[93] Cesarino I (2019). Structural features and regulation of lignin deposited upon biotic and abiotic stresses. Curr Opin Biotechnol.

[94] Baldassarini SFL, Esteves VLG, Ferreira RA, Lima MA, Moreira ND, Cláudia PA (2018). Proline accumulation induces the production of total phenolics in transgenic tobacco plants under water deficit without increasing the G6PDH activity. Theor Exp Plant Physiol.

[95] Liu W, Jiang Y, Wang C, Zhao L, Jin Y, Xing Q, Li M, Lv T, Qi H (2020). Lignin synthesized by CmCAD2 and CmCAD3 in oriental melon (Cucumis melo L.) seedlings contributes to drought tolerance. Plant Mol Biol..

[96] Chen K, Song M, Guo Y, Liu L, Xue H, Dai H, Zhang Z (2019). MdMYB46 could enhance salt and osmotic stress tolerance in apple by directly activating stress-responsive signals. Plant Biotechnol J.

[97] Zhang J, Yin XR, Li H, Xu M, Zhang MX, Li SJ, Liu XF, Shi YN, Grierson D, Chen KS (2020). Ethylene response factor 39-MYB8 complex regulates low-temperature-induced lignification of loquat fruit. J Exp Bot.

[98] Yuan L, Grotewold E (2020). Plant specialized metabolism. Plant Sci.

[99] Guan Y, Hu W, Jiang A, Xu Y, Sa R, Feng K, Zhao M, Yu J, Ji Y, Hou M, Yang X (2019). Effect of methyl jasmonate on phenolic accumulation in wounded broccoli. Molecules.

[100] Zhu Z, An F, Feng Y, Li P, Xue L, A M, Jiang Z, Kim JM, To TK, Li W, Zhang X, Yu Q, Dong Z, Chen WQ, Seki M, Zhou JM, Guo H (2011). Derepression of ethylene-stabilized transcription factors (EIN3/EIL1) mediates jasmonate and ethylene signaling synergy in Arabidopsis. Proc Natl Acad Sci USA.

[101] Taki N, Sasaki-Sekimoto Y, Obayashi T, Kikuta A, Kobayashi K, Ainai T, Yagi K, Sakurai N, Suzuki H, Masuda T, Takamiya K, Shibata D, Kobayashi Y, Ohta H (2005). 12-oxo-Phytodienoic acid triggers expression of a distinct set of genes and plays a role in wound-induced gene expression in Arabidopsis. Plant Physiol.

[102] Meng L, Yang Y, Ma Z, Jiang J, Zhang X, Chen Z, Cui G, Yin X (2022). Integrated physiological, transcriptomic and metabolomic analysis of the response of Trifolium pratense L. to Pb toxicity. J Hazard Mater..

[103] Anwar A, She M, Wang K, Riaz B, Ye X (2018). Biological roles of ornithine aminotransferase (OAT) in plant stress tolerance: Present Progress and Future Perspectives. Int J Mol Sci.

[104] Singh S, Parihar P, Singh R, Singh VP, Prasad SM (2016). Heavy metal tolerance in plants: role of transcriptomics, proteomics, metabolomics, and ionomics. Front Plant Sci.

[105] Xu X, Legay S, Sergeant K, Zorzan S, Leclercq CC, Charton S, Giarola V, Liu X, Challabathula D, Renaut J, Hausman JF, Bartels D, Guerriero G (2021). Molecular insights into plant desiccation tolerance: transcriptomics, proteomics and targeted metabolite profiling in Craterostigma plantagineum. Plant J.

[106] Livingston DP, Hincha DK, Heyer AG (2009). Fructan and its relationship to abiotic stress tolerance in plants. Cell Mol Life Sci.

[107] Parvanova D, Ivanov S, Konstantinova T, Karanov E, Atanassov A, Tsvetkov T, Alexieva V, Djilianov D (2004). Transgenic tobacco plants accumulating osmolytes show reduced oxidative damage under freezing stress. Plant Physiol Biochem.

[108] Poulsen LR, López-Marqués RL, Pedas PR, McDowell SC, Brown E, Kunze R, Harper JF, Pomorski TG, Palmgren M (2015). A phospholipid uptake system in the model plant Arabidopsis thaliana. Nat Commun.

[109] Vigh L, Maresca B, Harwood JL (1998). Does the membrane’s physical state control the expression of heat shock and other genes?. Trends Biochem Sci.

[110] Schmittgen TD, Livak KJ (2008). Analyzing real-time PCR data by the comparative C(T) method. Nat Protoc.

[111] Shahmuradov I, Solovyev V (2015). Nsite, NsiteH and NsiteM computer tools for studying transcription regulatory elements. Bioinform.

[112] Saito R, Smoot ME, Ono K, Ruscheinski J, Wang PL, Lotia S, Pico A, Bader DG, Ideker T (2012). A travel guide to Cytoscape plugins. Nat Methods.

