# Supplementary material for: Integrated analyses reveal the response of peanut to phosphorus deficiency on phenotype, transcriptome and metabolome
Source: BMC Plant Biol. 2022 Nov 14;22:524. doi: 10.1186/s12870-022-03867-4 (PMC9661748; doi:10.1186/s12870-022-03867-4)
Supplement: Supplementary file 3 — Additional file 3: Supplemental Fig. S1. Effect of phosphorus on activities of related enzymes and hormone in peanut leaves. * indicates P < 0.05. ** indicates P < 0.01. Supplemental Fig. S2. Differentially expressed gene (DEGs) clustering. (A) Heat map of differentially expressed gene. Transformed TPM (Transcripts Per Million reads) values were used to measure expression level. Red color represents the high expression level; and blue color represents the low expression level. C1-C3 indicates +P group; T1-T3 indicates –P group. (B) The red dots indicates the significantly up-regulated genes, and the green dots indicates the significantly down-regulated genes. Supplemental Fig. S3. Verification and consistency analysis of the selected genes expression level between RNA-Seq and qRT-PCR results in peanut roots. Supplemental Fig. S4. Sequence lengths distribution of obtained miRNAs by high throughput sequencing in each sample. C1-C3 indicates +P group; T1-T3 indicates –P group. Supplemental Fig. S5. Top15 expressed miRNAs (A) and expression distribution (B) in each sample. Supplemental Fig. S6. qRT-PCR verification and consistency analysis of the selected miRNAs and target genes in peanut roots. Supplemental Fig. S7. OPLS-DA analysis (A) and heat map of DAMs clustering. C1-C3 indicates +P group; T1-T3 indicates –P group. Supplemental Fig. S8. Differentially accumulated metabolites (DAMs) between treatments. (A) Volcano plot showing the DAMs between two P levels. The red dots indicates the significantly increased DAMs, the green dots indicates the significantly decreased DAMs. (B) PCA analysis of samples. (C) Correlation analysis between samples. (D) Correlation analysis of DAMs. Supplemental Fig. S9. Compounds classification and function analysis. (A) Compounds classification by KEGG Compound database. (B) Compounds classification by HMDB database. (C) KEGG pathway classification. (D) KEGG enrichment analysis of DAMs. [file 12870_2022_3867_MOESM3_ESM.docx]

Fig. S1


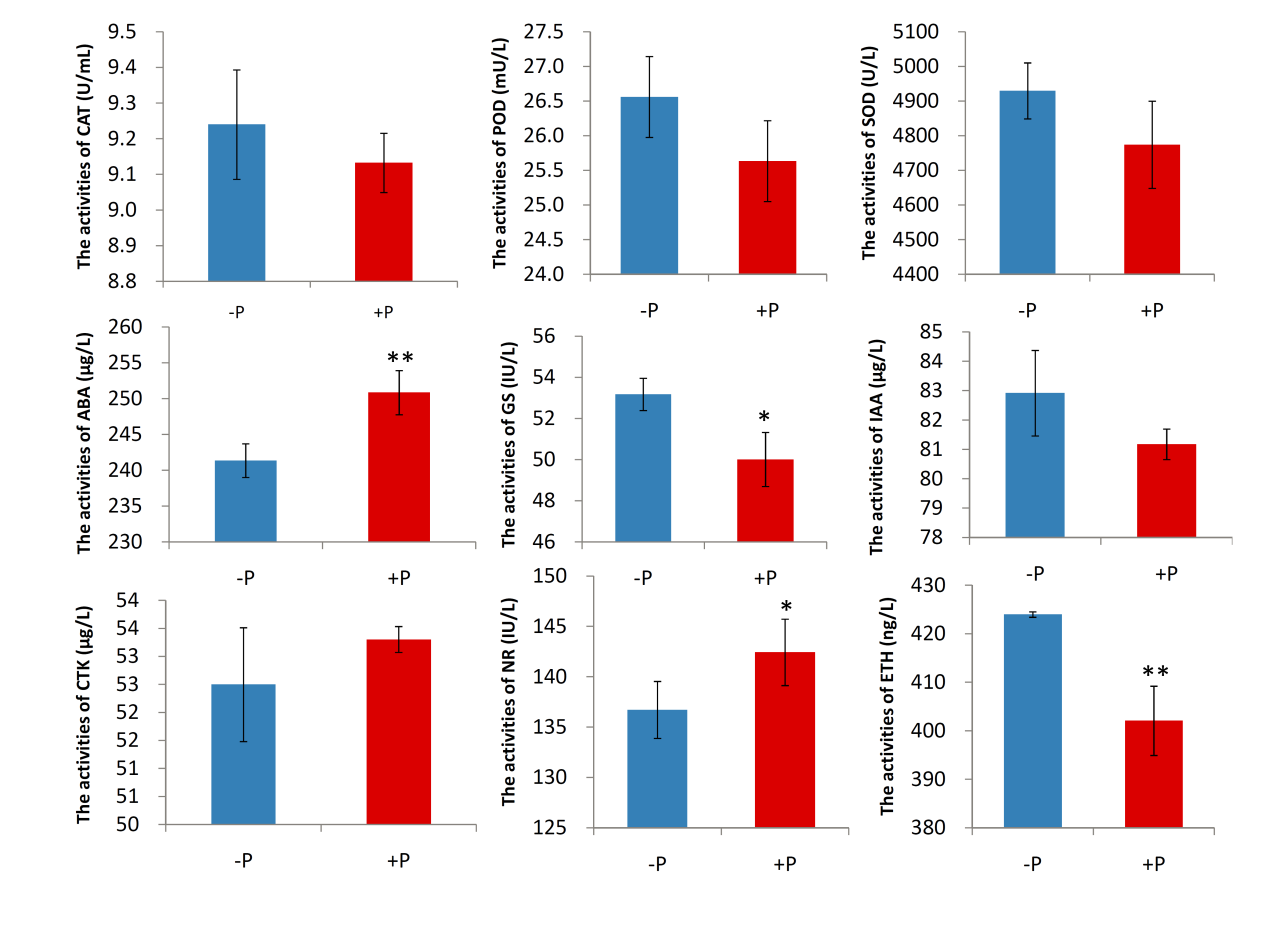


**Supplemental Fig. S1** Effect of phosphorus on activities of related enzymes and hormone in peanut leaves. * indicates *P* < 0.05. ** indicates *P* < 0.01.

Fig. S2


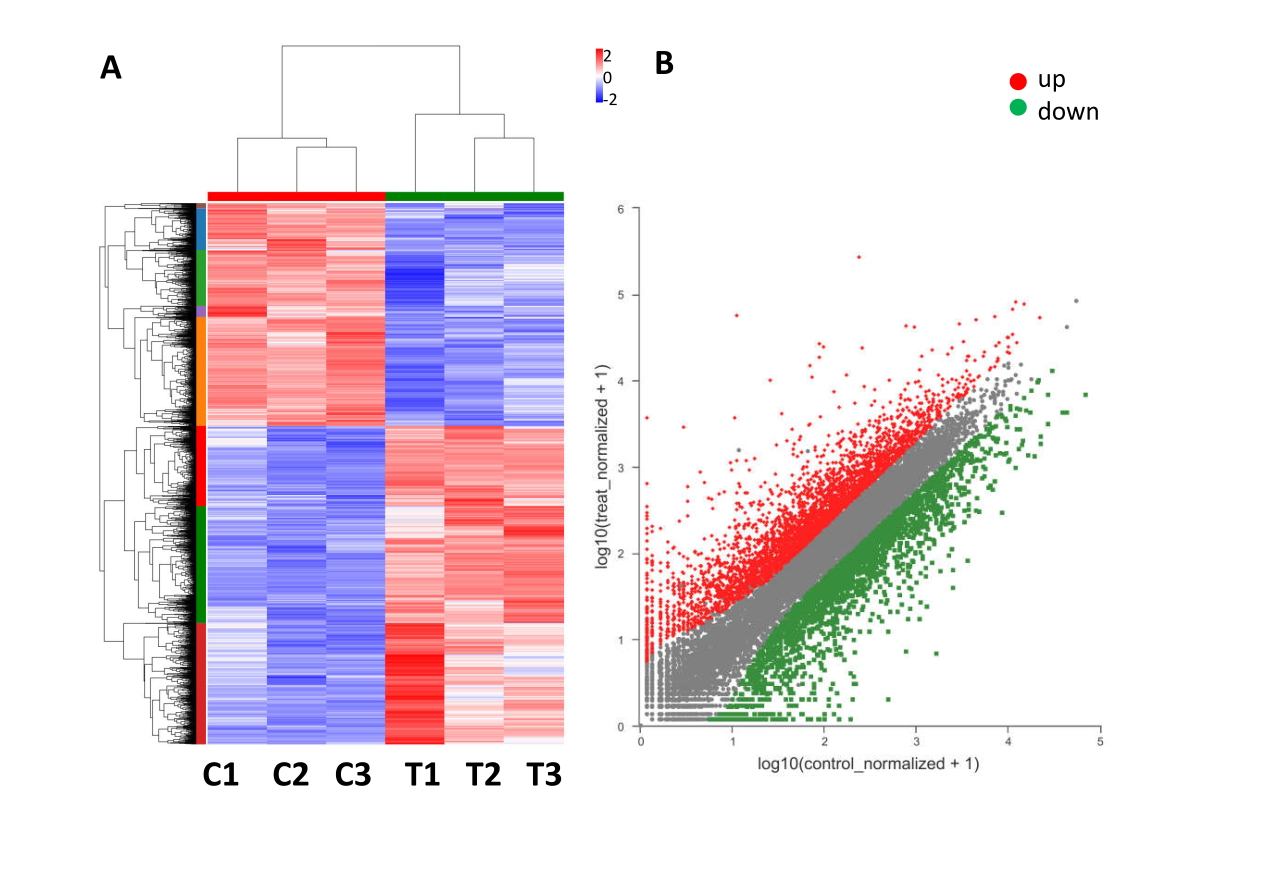


**Supplemental Fig.** **S2** Differentially expressed gene (DEGs) clustering. (A) Heat map of differentially expressed gene. Transformed TPM (Transcripts Per Million reads) values were used to measure expression level. Red color represents the high expression level; and blue color represents the low expression level. C1-C3 indicates +P group; T1-T3 indicates –P group. (B) The red dots indicates the significantly up-regulated genes, and the green dots indicates the significantly down regulated genes.

Fig. S3


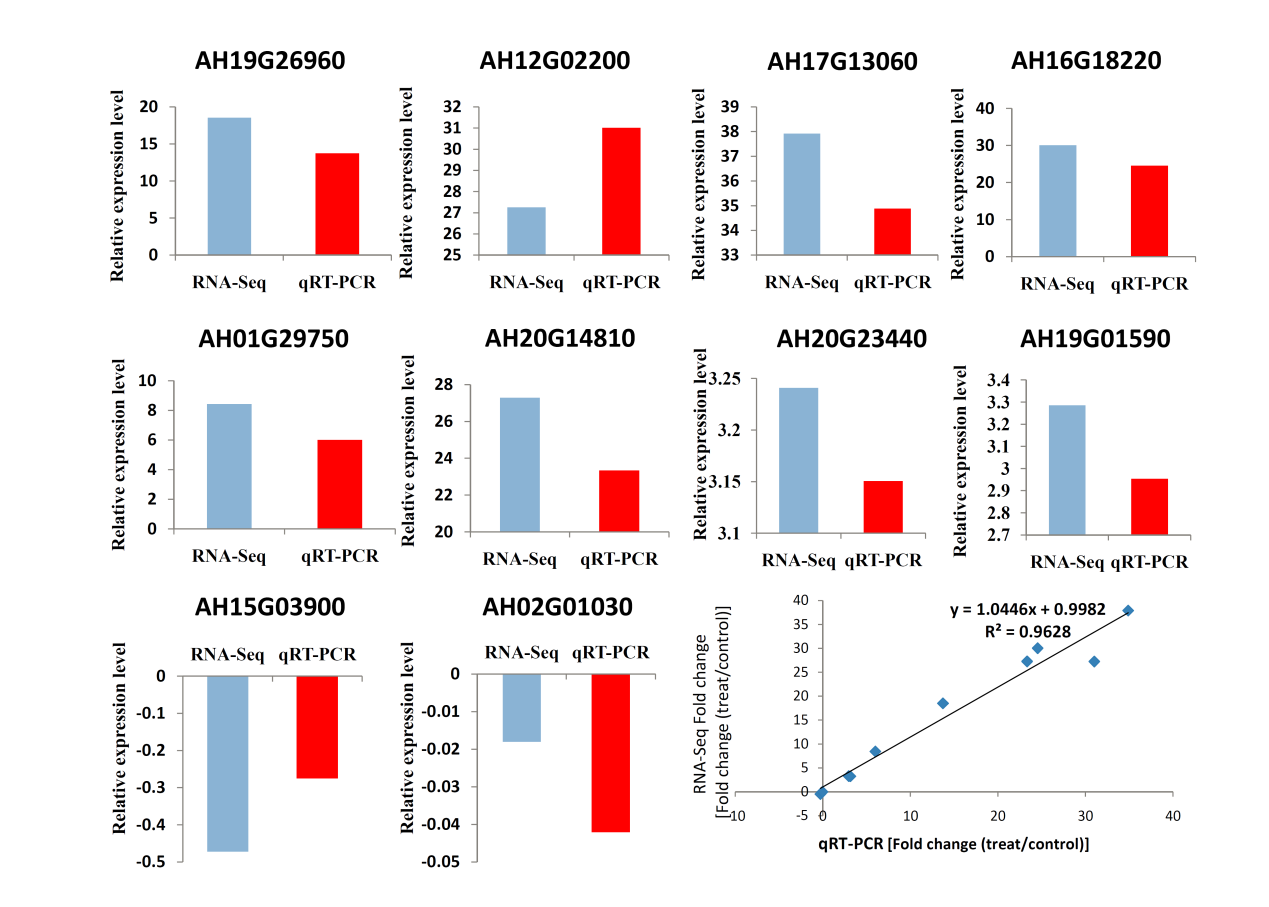


**Supplemental Fig.** **S3** Verification and consistency analysis of the selected genes expression level between RNA-Seq and qRT-PCR results in peanut roots.

Fig. S4


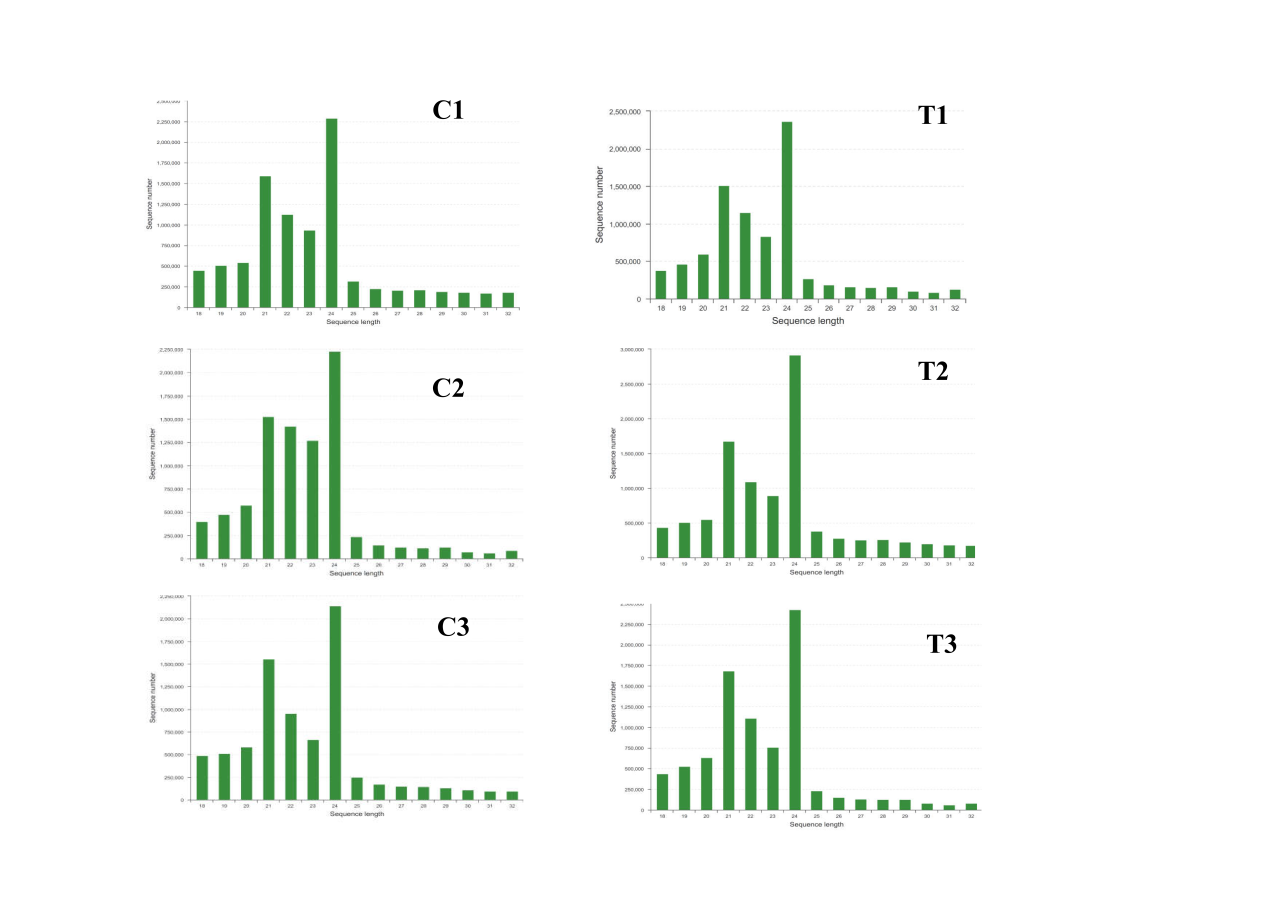


**Supplemental Fig.** **S4** Sequence lengths distribution of obtained miRNAs by high throughput sequencing in each sample. C1-C3 indicates +P group; T1-T3 indicates –P group.

Fig. S5


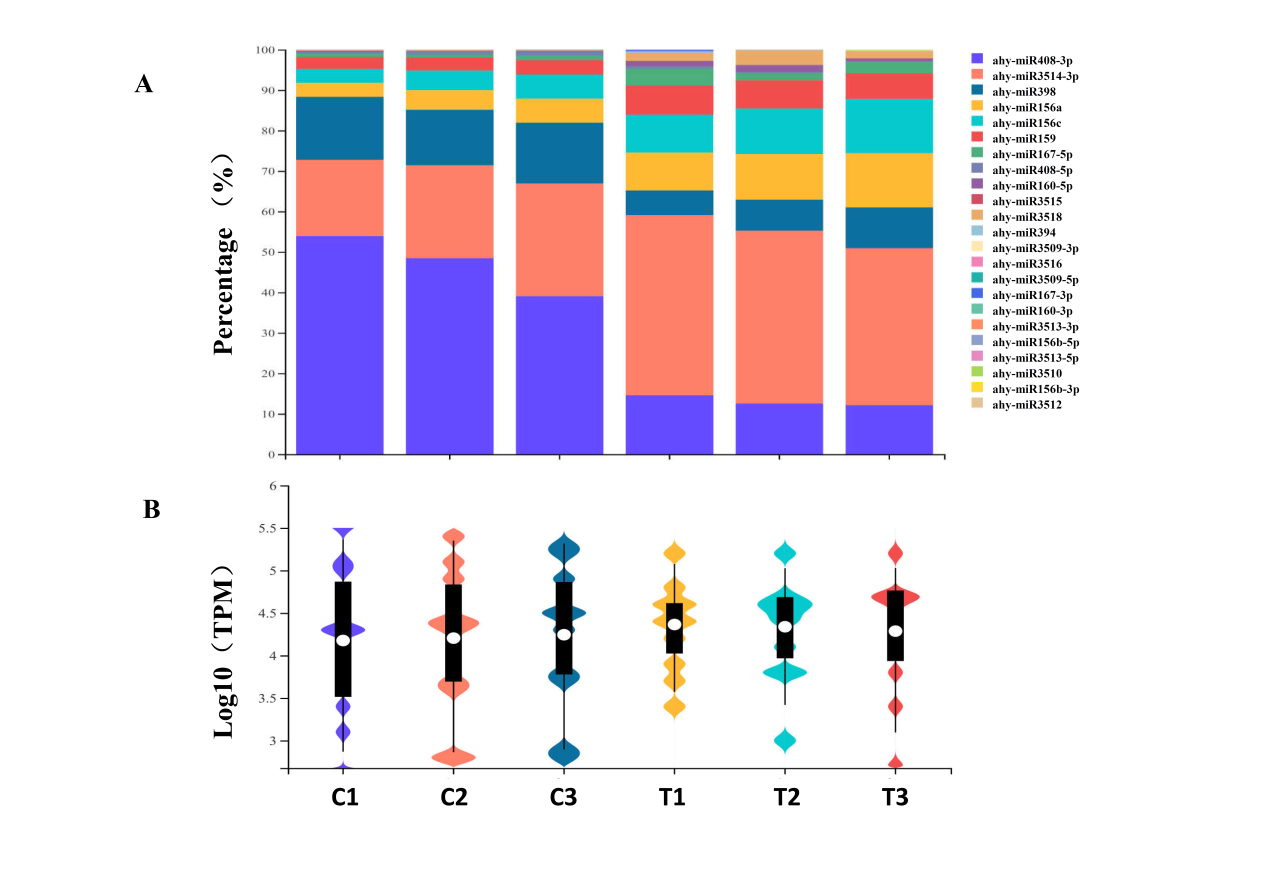


**Supplemental Fig.** **S5** Top15 expressed miRNAs (A) and expression distribution (B) in each sample.

Fig. S6


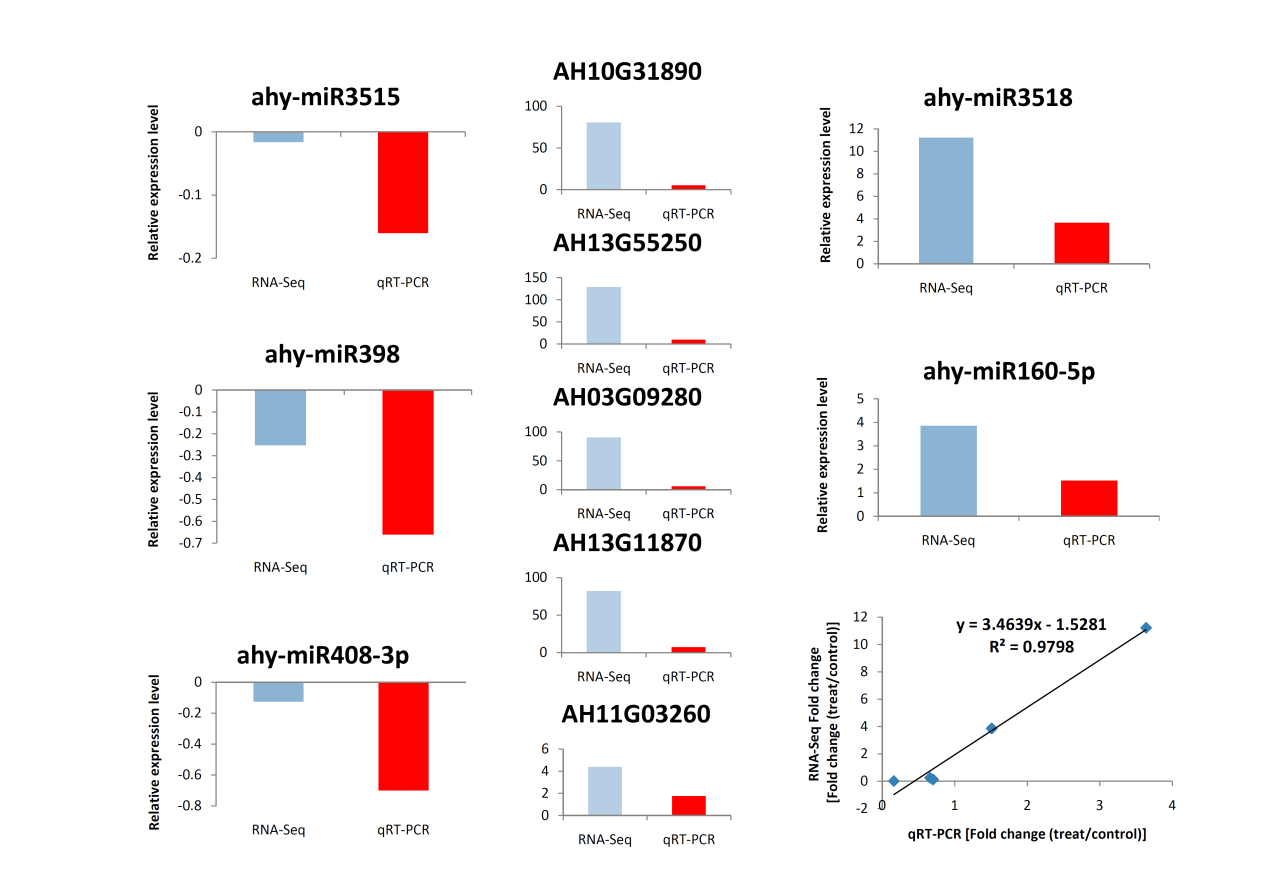


**Supplemental Fig.** **S6** qRT-PCR verification and consistency analysis of the selected miRNAs and target genes in peanut roots.

Fig. S7


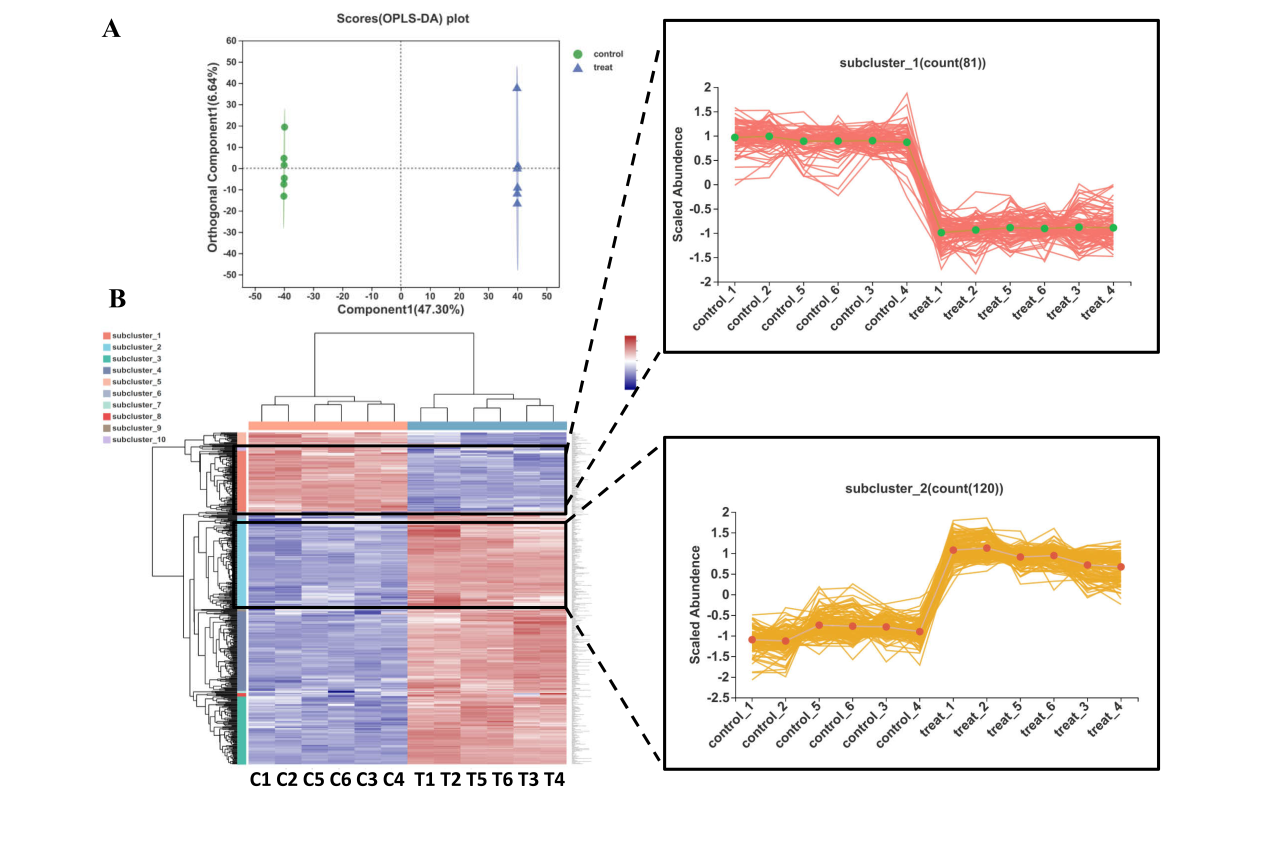


**Supplemental Fig.** **S7** OPLS-DA analysis (A) and heat map of DAMs clustering. C1-C3 indicates +P group; T1-T3 indicates –P group.

Fig. S8


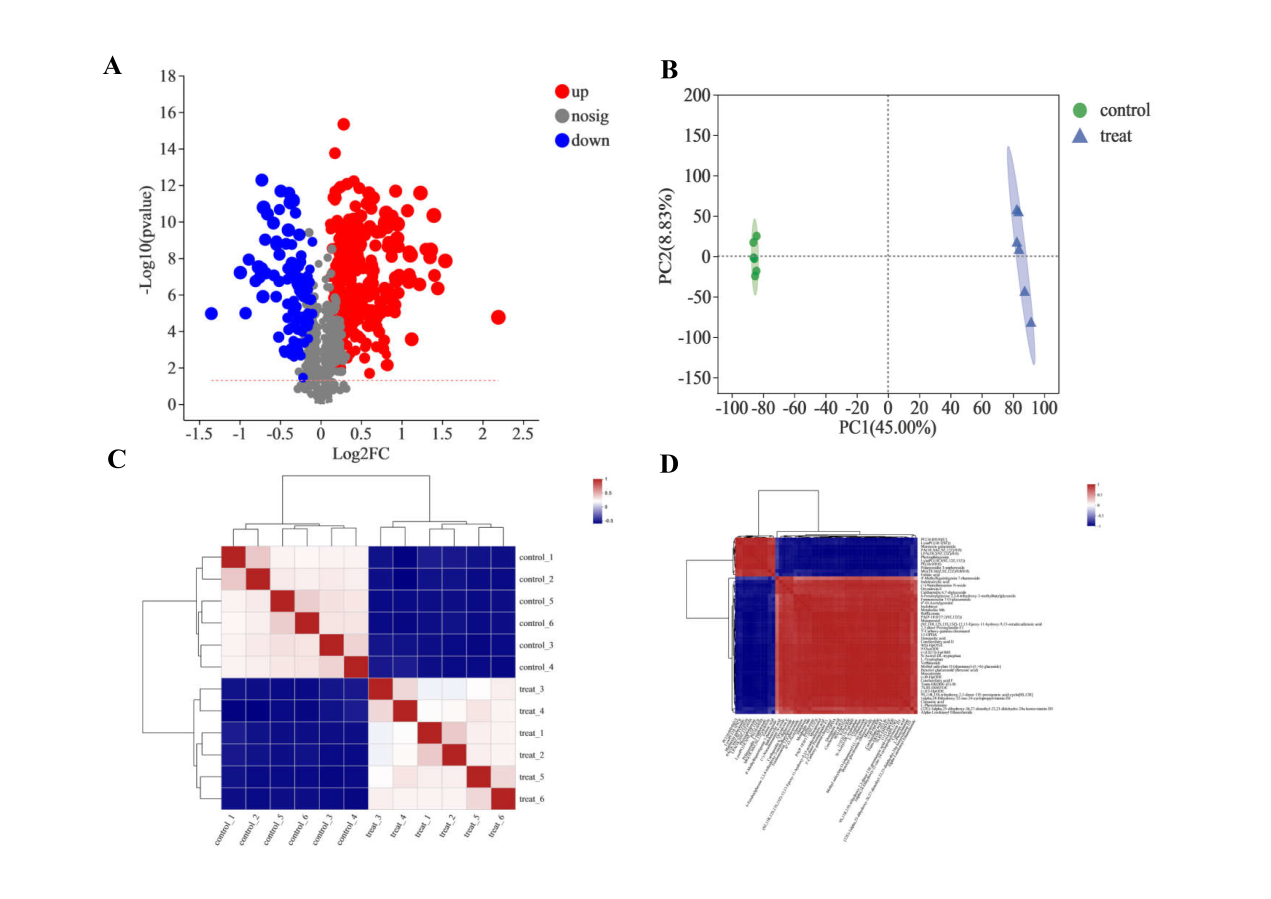


**Supplemental Fig.** **S8** Differentially accumulated metabolites (DAMs) between treatments. (A) Volcano plot showing the DAMs between two P levels. The red dots indicates the significantly increased DAMs, the green dots indicates the significantly decreased DAMs. (B) PCA analysis of samples. (C) Correlation analysis between samples. (D) Correlation analysis of DAMs.

Fig. S9


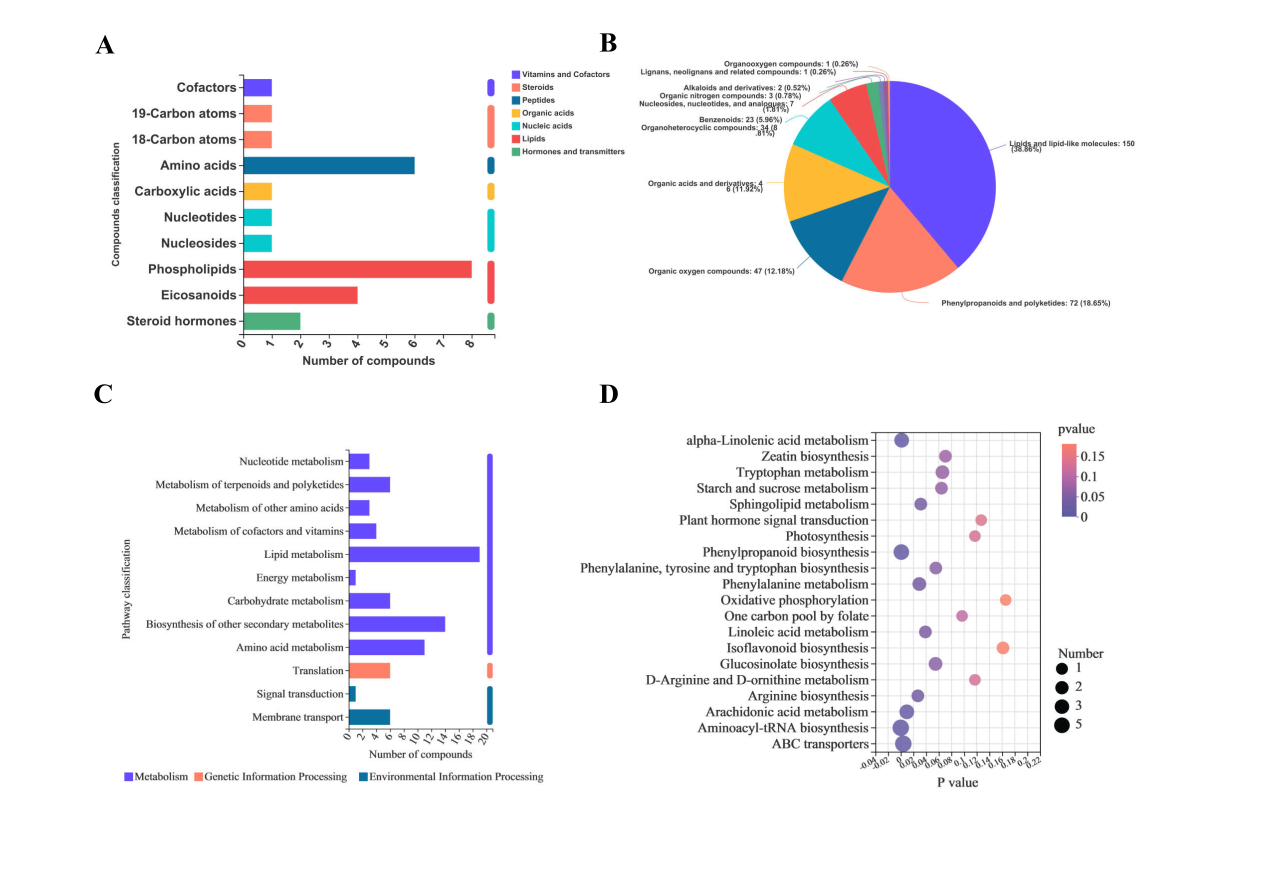


**Supplemental Fig.** **S9** Compounds classification and function analysis. (A) Compounds classification by KEGG Compound database. (B) Compounds classification by HMDB database. (C) KEGG pathway classification. (D) KEGG enrichment analysis of DAMs.
